# An evaluation of GPT models for phenotype concept recognition

**DOI:** 10.1186/s12911-024-02439-w

**Published:** 2024-01-31

**Authors:** Tudor Groza, Harry Caufield, Dylan Gration, Gareth Baynam, Melissa A. Haendel, Peter N. Robinson, Christopher J. Mungall, Justin T. Reese

**Affiliations:** 1 Rare Care Centre, Perth Children’s Hospital, 15 Hospital Avenue, Nedlands, WA 6009 Australia; 2 Telethon Kids Institute, 15 Hospital Avenue, Nedlands, WA 6009 Australia; 3 School of Electrical Engineering, Computing and Mathematical Sciences, Curtin University, Kent St, Bentley, WA 6102 Australia; 4 SingHealth Duke-NUS Institute of Precision Medicine, 5 Hospital Drive Level 9, Singapore, 169609 Singapore; 5 Division of Environmental Genomics and Systems Biology, Lawrence Berkeley National Laboratory, Berkeley, CA 94720 USA; 6 Western Australian Register of Developmental Anomalies, King Edward Memorial Hospital, 374 Bagot Road, Subiaco, WA 6008 Australia; 7 Faculty of Health and Medical Sciences, University of Western Australia, 35 Stirling Hwy, Crawley, WA 6009 Australia; 8 University of Colorado Anschutz Medical Campus, Aurora, CO 80045 USA; 9 The Jackson Laboratory for Genomic Medicine, Farmington, CT 06032 USA; 10 Institute for Systems Genomics, University of Connecticut, Farmington, CT 06032 USA

**Keywords:** Large language models, Generative pretrained transformer, Artificial intelligence, Phenotype concept recognition, Human Phenotype Ontology

## Abstract

**Objective:**

Clinical deep phenotyping and phenotype annotation play a critical role in both the diagnosis of patients with rare disorders as well as in building computationally-tractable knowledge in the rare disorders field. These processes rely on using ontology concepts, often from the Human Phenotype Ontology, in conjunction with a phenotype concept recognition task (supported usually by machine learning methods) to curate patient profiles or existing scientific literature. With the significant shift in the use of large language models (LLMs) for most NLP tasks, we examine the performance of the latest Generative Pre-trained Transformer (GPT) models underpinning ChatGPT as a foundation for the tasks of clinical phenotyping and phenotype annotation.

**Materials and methods:**

The experimental setup of the study included seven prompts of various levels of specificity, two GPT models (gpt-3.5-turbo and gpt-4.0) and two established gold standard corpora for phenotype recognition, one consisting of publication abstracts and the other clinical observations.

**Results:**

The best run, using in-context learning, achieved 0.58 document-level F1 score on publication abstracts and 0.75 document-level F1 score on clinical observations, as well as a mention-level F1 score of 0.7, which surpasses the current best in class tool. Without in-context learning, however, performance is significantly below the existing approaches.

**Conclusion:**

Our experiments show that gpt-4.0 surpasses the state of the art performance if the task is constrained to a subset of the target ontology where there is prior knowledge of the terms that are expected to be matched. While the results are promising, the non-deterministic nature of the outcomes, the high cost and the lack of concordance between different runs using the same prompt and input make the use of these LLMs challenging for this particular task.

**Supplementary Information:**

The online version contains supplementary material available at 10.1186/s12911-024-02439-w.

## Introduction

Over the past decade, clinical deep phenotyping—i.e., the comprehensive documentation of abnormal physical characteristics and traits in a computationally-tractable manner—has evolved into a common procedure for individuals who are either suspected of having or have been diagnosed with a rare disease. Similarly, the development and continuous enrichment of knowledge bases in the rare disease domain has become standard practice. Conceptually, both tasks rely on ontologies that are developed and updated by the medical community. Such ontologies facilitate the description of a patient's unique phenotype, as well as the characterisation of the phenotypic manifestations of gene mutations using ontological terms and concepts. The utility of using ontology-coded knowledge in rare diseases has been showcased repeatedly over the years in data sharing [1–3] and clinical variant prioritization and interpretation [4–6].

**Fig. 1:**
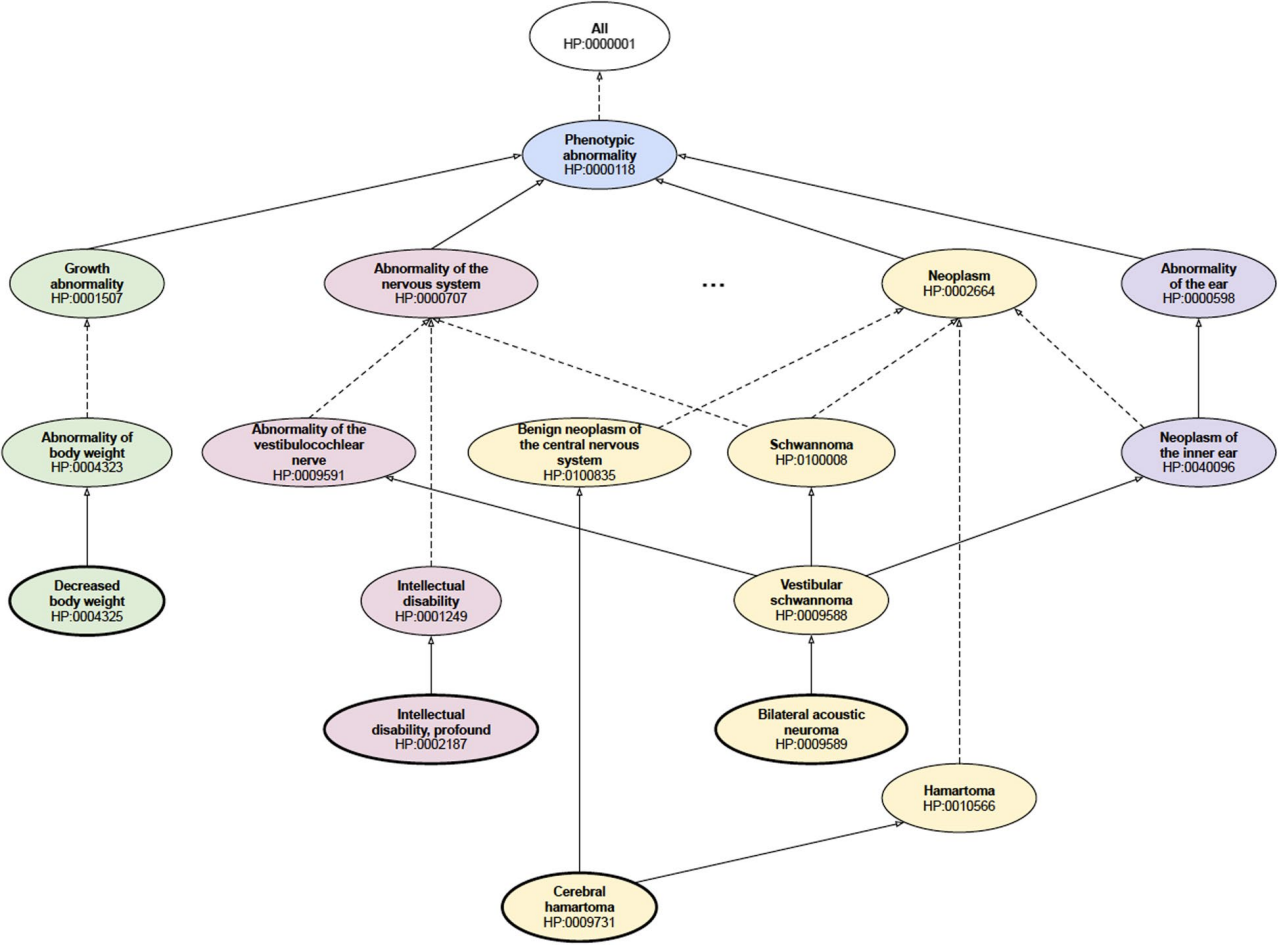
Simplified example of Human Phenotype Ontology concepts and their structural arrangement in the hierarchy. Solid lines denote direct parent–child relationships, while dotted lines denote ancestor–descendant relationships

The Human Phenotype Ontology (HPO) [7, 8] provides the most comprehensive resource for computational deep phenotyping and has become the de facto standard for encoding phenotypes in the rare disease domain, for both disease definitions as well as patient profiles to aid genomic diagnostics. The ontology, maintained by the Monarch Initiative [9], provides a set of more than 16,500 terms describing human phenotypic abnormalities, arranged as a hierarchy with the most specific terms furthest from the root, as depicted in Fig. 1.

In addition to underpinning complex diagnostic tasks (e.g., clinical interpretation of an exome/genome) [10] or building care coordination plans, ontology-coded phenotypes often represent also a communication channel between practitioners and patients and, subsequently, between patients and other stakeholders—e.g., education, disability or welfare workers. Moreover, concepts grounded in HPO provide the explainability required to improve the transparency of the decision-making process, which can then support communication and documentation.

Manual curation of phenotype profiles – and manual annotation as a general task – is, however, tedious and has represented the main blocker to wide-spread uptake of computational deep phenotyping on the clinical side and to keeping rare disease knowledge bases up to date. (Semi-) automated methods that rely on natural language processing (NLP) have been introduced to remove this blocker and have gradually become the standard *modus operandi*. Such methods, the latest built using convolutional neural networks [11] or transformer-based architectures [12], are also addressing a variety of challenges associated with phenotype concept recognition (CR) such as ambiguity, use of metaphorical expressions, as well as negation and complex or nested structures.

Lately, the focus has shifted to large language models (LLMs) for most NLP tasks. LLMs-a class of transformer-based models trained on trillions of words of diverse texts [13]-showcased superior capabilities in application domains such as chatbots and text prediction [14]. Their main advantage also stems from having the ability to use in-context learning to perform specific tasks, without the need for further training or fine-tuning [15], which replaces the “traditional” task-driven training of machine learning models [16]. gpt-3.5 and gpt-4.0 are examples of such LLMs that have witnessed a rapid general user adoption via the ChatGPT application, a chatbot fine-tuned for conversation-based interactions with humans. A user can “prompt” ChatGPT to perform a variety of tasks, with or without the need to provide examples to support them.

In the biomedical domain, several domain-specific models have been published-BioBERT [15], PubMedBERT [17] or BioGPT [18] – and shown to perform well on NLP tasks including relationship extraction (e.g., drug-drug interactions or drug-target interactions) and question answering. The experimental results also included comparisons against GPT-2.0, a predecessor of the current models powering ChatGPT. Lately, several studies have been published on the utility of using GPT models for annotation (in general) [19, 20] and few discussed the efficiency of such models on concept recognition tasks, in particular phenotype concept recognition. Note that ontology-based concept recognition implies a joint task of named entity recognition (i.e., finding entities on interest in a text and their corresponding boundaries) and entity linking (i.e., aligning the entities found in the text to concepts defined in a given ontology). Experiments documenting the accuracy on named entity recognition—with a focus on diseases and chemical entities—were documented by Chen et al. [21], with gpt-4.0 (+ one-shot learning) achieving a performance poorer than a fine-tuned PubmedBERT, yet significantly better that gpt-3.5.

This paper examines the ability of gpt-3.5 and gpt-4.0 to perform phenotype concept recognition using HPO as a background ontology. Three different approaches are used to generate prompts to gain a deeper understanding of the limitations in various scenarios. Specifically, the experimental setup targets direct concept recognition – i.e., named entity recognition followed by an alignment to HPO concepts and in-context learning.

## Materials and methods

The study uses two gold standard corpora available in the literature for phenotype concept recognition: (i) a corpus of 228 scientific abstracts collected from PubMed, initially annotated and published by Groza et al. [22], and subsequently refined by Lobo et al. [23] (named HPO-GS from here on); and (ii) the dev component of the corpus made available through Track 3 of BioCreative VIII (454 entries), focusing on extraction and normalization of phenotypes resulting from genetic diseases, based on dysmorphology physical examination [24] (named BIOC-GS from here on). An example of an entry is: “ABDOMEN: Small umbilical hernia. Mild distention. Soft.”

All experiments were conducted using these two corpora. HPO-GS covers 2,773 HPO term mentions and a total of 497 unique HPO IDs, with the minimum size of a document being 138 characters, the maximum size 2,417 characters and the average being ~ 500 characters. BIOC-GS covers 783 HPO term mentions and a total of 358 unique HPO IDs, with the minimum size of an entry being 13 characters, the maximum 225 characters and the average ~ 56 characters. Note that we chose the dev component of Track 3 because of its similarity in the number of unique HPO IDs and its profile (described below) to HPO-GS. We were unable to download the test component of Track 3 and hence the results reported here are not comparable to the results published by the Track’s organisers.

**Fig. 2:**
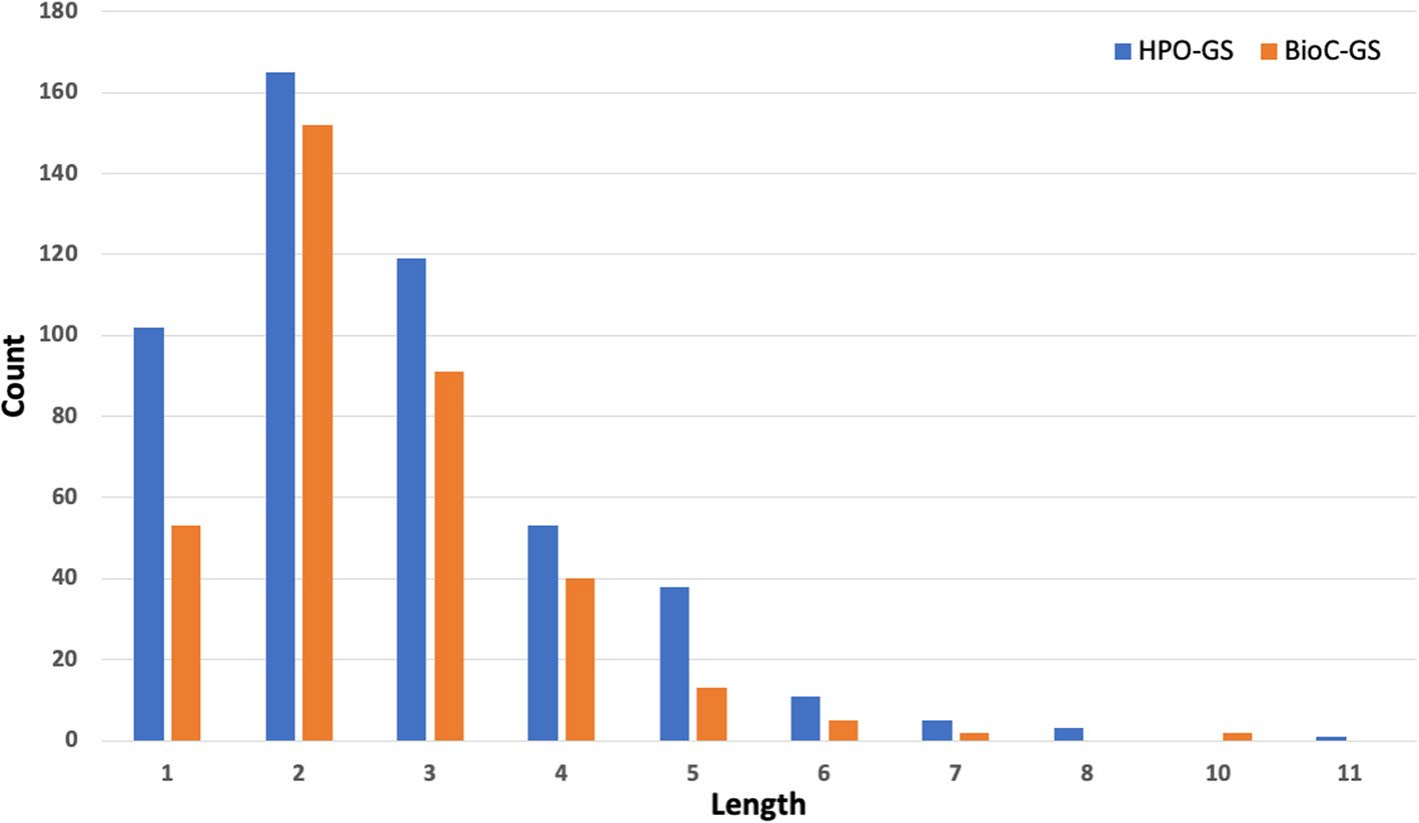
Label length distribution of the HPO concepts present in the gold standard corpus

**Fig. 3:**
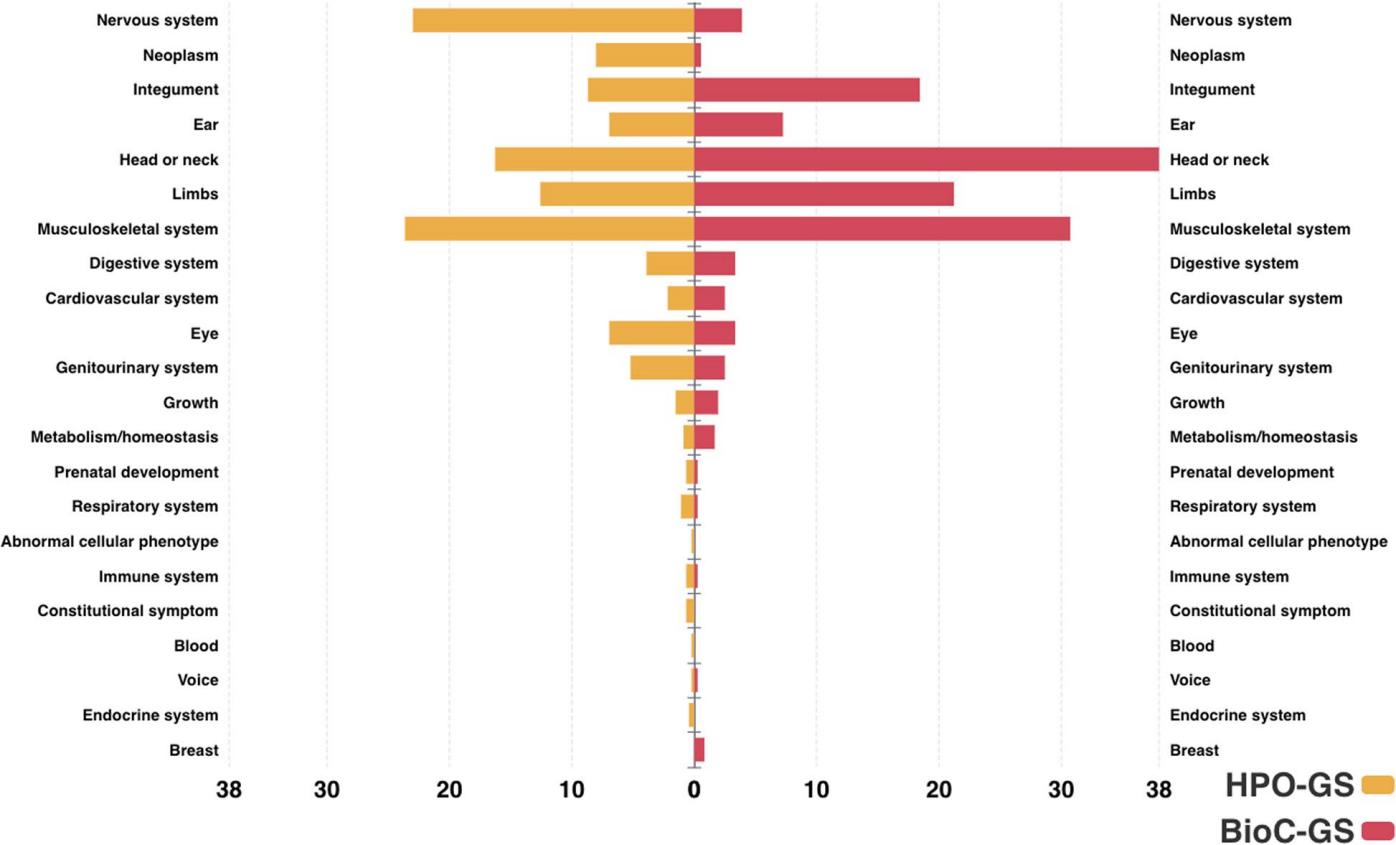
Top-level overview of the gold standard corpus using the children of ‘Phenotype abnormality’ as major categories

The complexity of the lexical representations of the HPO concepts can be partially assessed based on their length (presented in Fig. 2) and structural placement in the ontology. The latter is depicted in Fig. 3 using the children of the *Phenotypic abnormality* concept as major categories and the values representing the proportion of terms belonging to each category (as also depicted in Fig. 1). It can be observed that the large majority of concepts in both corpora (~ 88%) have low to moderate lexical complexity, with a label length of 4 words or less, and are placed predominantly in the nervous and musculoskeletal system (including here also head and neck and limbs)—i.e., denoting finger, toe, face, arm and leg abnormalities.

### Prompt generation approaches

Conversational models such as gpt-3.5-turbo and gpt-4.0 take inputs in the form of prompts. These prompts can include—in addition to a target content—explicit instructions or examples of the desired output. This is sometimes referred to as “prompt engineering”. A task, such as concept recognition, can be defined via prompts in various ways, with the behaviour and hence the output of the model being heavily influenced by smallest differences in these definitions. In this study, we used three types of prompts to investigate the models’ efficiency to perform phenotype concept recognition. Two remarks are worth noting about our selection strategy:

- We opted for well-known, low-barrier prompts that do not require significant prompt engineering knowledge and skills
- We were aware of the HPO ID hallucinations as a result of aiming for concept recognition – instead of a chain of entity recognition, external entity linking and LLM-based validation, however, as presented later in our experiments, this did not materialise as a real concern.

#### Instructional (directed) phenotype concept recognition

Prompts in this category aimed to capture the impact of the wording used to define ‘phenotypes’ on the CR task. They are instructional (directed) because the model is asked explicitly to perform a certain task. The four prompts defined in this category are listed below; the key instructions are underlined for easier comprehension.

- Prompt 1: *Analyze the text below delimited by triple backticks and extract phenotypes and clinical abnormalities. Align the phenotypes and clinical abnormalities found to Human Phenotype Ontology IDs. List the results in a JSON format using the following structure*.
- Prompt 2: *Analyze the text below delimited by triple backticks, extract phenotypes and align them to Human Phenotype Ontology IDs. List the results in a JSON format using the following structure. Where you cannot find a direct Human Phenotype Ontology ID, leave the “hpoId” field empty*.
- Prompt 3: *Analyze the text below delimited by triple backticks and extract Human Phenotype Ontology terms. List the HPO IDs together with the start and end offsets*.
- Prompt 4: *You will be provided with a text in triple backticks. The task is to perform automated concept recognition using the Human Phenotype Ontology and extract all Human Phenotype Ontology concepts found in the text. Include the HPO ID of the concepts you find in the result*.

The first three prompts direct the model to ‘extract’ artefacts from the provided text. Prompt 2 is a variation of Prompt 1 (‘phenotypes’ vs ‘phenotypes and clinical abnormalities’), while Prompt 3 refers directly to HPO terms. Prompt 4 explicitly names the task requested to be performed by the model—i.e., ‘automated concept recognition’.

#### Instructional (directed) named entity recognition followed by instructional (directed) entity alignment

The prompts in the first category target directly concept recognition by requesting HPO IDs. As a task, concept recognition can also be modelled as named entity recognition (used to detect entity boundaries in the text) followed by entity alignment (used to match the candidates extracted from the text to ontology concepts / IDs). Prompt sets 5 and 6 below explicitly perform this two-step process by first asking the model to extract phenotypes, then using this output as input to align the text to HPO IDs. Prompt set 6 is a subset of Prompt set 5 – ‘phenotypes’ vs ‘phenotypes and clinical abnormalities’.

- Prompt set 5:
  - ◦ Step 1: *Analyze the text below delimited by triple backticks and extract phenotypes and clinical abnormalities. List them together with the start and end offsets.*
  - ◦ Step 2: *You will be provided with text delimited by triple backticks. Align the text below to Human Phenotype Ontology labels. List only the HPO concepts found.*
- Prompt set 6:
  - ◦ Step 1: *Analyze the text below delimited by triple backticks and extract phenotypes. List them together with the start and end offsets in the text.*
  - ◦ Step 2: *You will be provided with text delimited by triple backticks. Align the text below to Human Phenotype Ontology labels. List only the HPO concepts found*.

#### In-context learning using a subset of HPO

The final category (prompt set 7) attempts to aid the model by providing examples of the concepts targeted for extraction. The prompt used a standard two-part template, as below:

- Part 1: Examples: The Human Phenotype Ontology defines phenotype concepts using the following label – HPO ID associations: *Hypospadias* // HP:0000047.
- Part 2: Task: Using the list above, find Human Phenotype Ontology concepts in the following text and return their associated IDs for every appearance in the text.

Part 1 was completed by adding the pairs of label—HPO ID for all HPO concepts present in the gold standard corpus. Attempts were made to include the entire ontology, or to include the labels and all synonyms for the desired HPO concepts, however they failed due to the model limitations on the size of the input content. We do, however, demonstrate the impact of using various sets of concepts to underpin the in-context learning task. A complete example of prompt 7 is provided in Appendix 1 in the Supplementary material.

### Experimental setup

Experiments were conducted by calling the GPT models using the OpenAI API (https://platform.openai.com/docs/api-reference). The specific models (as per https://platform.openai.com/docs/models/continuous-model-upgrades) and parameters used in our experiments were:

- gpt-3.5-turbo-16k – with training data “up to Sep 2021” and context window of 16,385 tokens
- gpt-4-1106-preview – with training data “up to Apr 2023” and context window of 128,000 tokens
- Temperature: 0
- Max tokens: default – as permitted by the model (note that the combination of the context window and default max tokens enabled us to experiment with Prompt 7)

Each call used one of the seven prompts discussed above and the text corresponding to each abstract or examination entry, individually, as user input. The results were stored individually and HPO concepts were extracted and associated with the PMID / entry ID corresponding to the text used as input. The code used to annotate the corpora and perform the evaluation is available at: https://github.com/tudorgroza/code-for-papers.

The standard evaluation procedure for concept recognition covers two aspects: (i) boundary detection—i.e., a correct alignment of the boundaries of the concepts in text, usually by matching the offsets of the corresponding text span to the offsets found by the system being evaluated; and (ii) concept mapping—i.e., a correct matching of the ID of the concept against that provided by the gold standard. The boundary detection step proved to be challenging to evaluate accurately with the results produced by the OpenAI GPT models – an aspect documented also by Chen et al. [19]. Consequently, given our focus on understanding the utility of these models to support manual phenotype annotation / curation, we relaxed the evaluation procedure to include only the second step—i.e., concept mapping. A correct match was, therefore, counted if the HPO ID present in the gold standard was found at least once by the LLM.

The evaluation metrics used in this experiment are the standard for the task: precision, recall and F1. These were computed at both document and mention levels. The document-level defines a true positive when a desired HPO ID is found at least once by the LLM, while the mention-level keeps track of all encounters of the HPO ID in a particular abstract and defines a true positive when each individual encounter is found by the LLM.

The experimental results produced by the models are compared against five well-established phenotype concept recognition tools:

- Doc2HPO [25] – dictionary-based – API documented at https://github.com/stormliucong/doc2hpo
- ClinPheno [26] – dictionary-based – MacOS download version available on the 10th of August 2022 from http://bejerano.stanford.edu/clinphen/
- NCBO Annotator [27] – dictionary-based – API documented at https://bioportal.bioontology.org/annotator (default parameters)
- Monarch Annotator [9] – dictionary-based – API documented at https://monarchinitiative.org/ (default parameters; match over 5 characters long)
- PhenoTagger [12] – hybrid method combining dictionary tagging with a BioBERT-based tagger – release v1.1 downloaded from https://github.com/ncbi-nlp/PhenoTagger with models v1.1 downloaded from https://ftp.ncbi.nlm.nih.gov/pub/lu/PhenoTagger/models_v1.1.zip (executed with default parameters)

## Results

### Experimental results

Tables 1 and 2 list the experimental results achieved by both models across all seven prompts on HPO-GS and BIOC-GS respectively, while Table 3 lists the results of the state of the art methods for phenotype concept recognition. Below we discuss the main findings emerging from these results:

- *The in-context learning strategy achieves results comparable or better than the state of the art*. Phenotype concept recognition is known to be a difficult task—as showcased by the F1 scores listed in Table 3, which are roughly 0.2 lower that other domain-specific concept recognition tasks, such as gene or drug names. The GPT models perform significantly lower that the state of the art in most cases, with the mention-level evaluation F1 scores being half the values of tools such as PhenoTagger or the Monarch Annotator (which does not rely on a BERT-based or a LLM-based on neural network-based architecture). The in-context learning strategy, however, performs well; HPO-GS the results are comparable to the state of the art, on BIOC-GS gpt-4.0 surpasses the best in class with a significant margin – almost 0.1 (0.7 F1 on mention-level evaluation compared to 0.61 F1 for PhenoTagger). Although this strategy would not serve phenotype concept recognition in general, it would support manual annotation in a clearly defined domain—e.g., cardiovascular diseases.
- *Both models have a consistent behaviour across prompts*. Prompts 1 and 2—defining the task as an extraction of phenotypes and clinical abnormalities, followed by an alignment to HPO IDs—achieve the best precision, with Prompt 2 leading to better results when using gpt-3.5 (although not gpt-4.0). Similarly, Prompt 7 (in-context learning) achieved the best recall, which was expected since the examples included all concepts present in the gold standard.
- *Document and mention-level evaluation results show significant discrepancies*. The mention-level experimental results were surprisingly lower than the document-level results on HPO-GS. This could be attributed to the variability of the lexical representations of the concepts in text—e.g., *Brachydactyly C* vs *Brachydactyly, type C.* This outcome does not hold on BIOC-GS, which seems to be more uniform.

**Table 1:** Document and mention-level evaluation results across both models and all seven prompts on HPO-GS

GPT version	Prompt	Precision	Recall	F1	Precision	Recall	F1
		**Document-level**			**Mention-level**		
3.5	1	0.45	0.21	0.29	0.39	0.14	0.20
	2	**0.51**	0.12	0.19	**0.46**	0.08	0.13
	3	0.12	0.25	0.16	0.05	0.15	0.07
	4	0.12	0.28	0.16	0.07	0.17	0.10
	5	0.14	0.09	0.11	0.14	0.06	0.08
	6	0.3	0.13	0.18	0.29	0.08	0.12
	7	0.41	**0.41**	**0.41**	0.28	**0.25**	**0.26**
4	1	0.41	0.34	0.37	0.36	0.21	0.26
	2	0.41	0.34	0.37	0.36	0.21	0.26
	3	0.37	0.31	0.33	0.34	0.19	0.24
	4	0.34	0.38	0.35	0.32	0.23	**0.27**
	5	0.31	0.22	0.25	0.26	0.13	0.17
	6	0.35	0.17	0.22	0.29	0.10	0.15
	7	**0.75**	**0.47**	**0.58**	**0.73**	**0.3**	**0.43**

**Table 2:** Document and mention-level evaluation results across both models and all seven prompts on BIOC-GS

GPT version	Prompt	Precision	Recall	F1	Precision	Recall	F1
		**Document-level**			**Mention-level**		
3.5	1	0.51	0.12	0.19	0.5	0.11	0.18
	2	**0.68**	0.05	0.09	**0.68**	0.05	0.09
	3	0.27	0.29	0.28	0.26	0.25	0.25
	4	0.26	0.33	0.29	0.22	0.29	0.25
	5	0.31	0.2	0.24	0.3	0.17	0.22
	6	0.31	0.2	0.24	0.3	0.17	0.22
	7	0.56	**0.56**	**0.56**	0.54	**0.49**	**0.51**
4	1	0.46	0.44	0.45	0.45	0.39	0.42
	2	0.44	0.44	0.44	0.43	0.38	0.4
	3	0.47	0.43	0.45	0.47	0.37	0.41
	4	0.43	0.53	0.47	0.43	0.46	0.44
	5	0.44	0.27	0.33	0.43	0.24	0.31
	6	0.44	0.27	0.33	0.43	0.24	0.31
	7	**0.78**	**0.73**	**0.75**	**0.77**	**0.64**	**0.7**

**Table 3:** Mention-level evaluation results of the state-of-the-art methods for phenotype concept recognition and for reference purposes the results of the best-performing GPT models

	**HPO-GS**			**BioC-GS**		
Tool	**Precision**	**Recall**	**F1**	**Precision**	**Recall**	**F1**
PhenoTagger [12]	0.77	**0.68**	**0.72**	0.74	0.52	**0.61**
ClinPheno [26]	0.73	0.36	0.48	0.47	**0.57**	0.52
Doc2HPO [25]	0.81	0.50	0.62	**0.84**	0.29	0.43
Monarch Annotator [9]	**0.82**	0.50	0.62	0.47	0.46	0.46
NCBO Annotator [27]	0.66	0.49	0.56	0.78	0.41	0.54
Best non in-contex learning gpt (gpt-4, Prompt 4)	0.32	0.23	0.27	0.43	0.46	0.44
Best gpt-4 (Prompt 7; in-context learning)	0.73	0.3	0.43	0.77	**0.64**	**0.7**
Best gpt-3.5 (Prompt 7; in-context learning)	0.28	0.25	0.26	0.54	0.49	0.51

### LLAMA2-70B experimental results

To provide a reference comparison against a second large language model, we performed a full set of experiments using the freely available LLAMA2-70B model, provided by Meta (https://huggingface.co/docs/transformers/model_doc/llama2). An inference endpoint was setup via Anyscale (https://app.endpoints.anyscale.com/) and all prompts were executed on both corpora using the same values for the temperature (0) and max tokens (default) parameters. In-context learning strategy (Prompt 7) could not be used with LLAMA2-70B since the context window was significantly smaller (4096 tokens). The raw outcomes of the experiments are available at https://github.com/tudorgroza/code-for-papers/tree/main/gpt-pheno-cr/experiments/llama2-70B.

The performance of LLAMA2-70B was poor, with efficiency metrics (at both document and mention levels) ranging between 0 and 0.01. Remarkably, the model returned a mixture of terminologies (HPO IDs, UMLS CUIs, and ICD codes). We can, hence, conclude that LLAMA2 is not currently fit for HPO-based phenotype recognition without more in-depth analysis of complex prompting techniques.

### Hallucinations

Our experiments defined standard, community-accepted phenotype concept recognition tasks and the evaluation targeted HPO IDs extracted by the models. Hence, in terms of hallucinations.

(inaccurate, nonsensical, or irrelevant output given the given context), the expectation was to find non-existing HPO IDs in the output produced by the models. We observed an insignificant number of hallucinations (Table 4), and as such, hallucinations do not pose a challenge for this task. Some examples of hallucinations include: HP:0020115, HP:0025111, HP:0023656, HP:0031966, HP:0020019, HP:0040060. A second observation can be made with respect to Prompts 3 and 4 (instructing the model to perform the task by its name): these prompts are very prolific on HPO-GS (7780 HPO IDs found, and 6698, respectively), which leads to an increased recall and a lower precision.

**Table 4:** Overview of number of HPO IDs found in all experiments and associated hallucinations

Model	Prompt	Total found	Unique	Hall’s	Hall’s (%)	Total found	Unique	Hall’s	Hall’s (%)
**BASE**		**2,773**	**497**			783	358		
3.5	1	978	408	0	0	167	126	0	0
	2	460	237	0	0	53	45	0	0
	3	7780	1546	11	1	755	432	0	0
	4	6698	1980	14	1	996	484	0	0
	5	1095	841	3	0	351	242	0	0
	6	771	491	4	1	442	316	0	0
	7	2551	681	2	0	709	319	1	0
4	1	1617	634	1	0	666	390	0	0
	2	1605	633	2	0	699	397	0	0
	3	1534	728	2	0	625	366	1	0
	4	2003	855	7	1	839	479	3	1
	5	1408	636	4	1	1287	648	3	0
	6	977	483	4	1	432	288	2	1
	7	3469	938	0	0	676	313	1	0

False positives produced by the model are measured via precision in Tables 1 and 2, and could also be considered hallucinations. Given the nature of the underpinning task, however, a complex challenge emerges in distinguishing “real” false positives from hallucinations. A metric addressing this specific need could be devised, for example, by measuring the semantic distance between the correct HPO ID and the one found by the model.

### In-context learning with different sets of concepts

The results for Prompt 7 above relied on the same set of concepts as those present in the gold standard. To test the impact of this set on the results (which in a standard setting would be expected, since the entire ontology would be considered), we performed three additional experiments using gpt-4.0 and Prompt 7. Firstly, we used the concepts covered by HPO-GS to do in-context learning for BIOC-GS. The results were significantly lower, with the model achieving document-level precision, recall and F1 of 0.25, 0.23, 0.24 respectively and mention-level metrics of 0.23, 0.2, 0.21.

Secondly, we used the top-level profile of the two corpora (depicted in Fig. 3; i.e., the majority of the concepts describing musculo-skeletal abnormalities) to generate a random set of ~ 1,000 concepts. This resulted in a set comprising 1,165 HPO concepts (~ 43KB in size with labels and ~ 7% of the entire ontology) and the following overlaps with the two gold standard corpora: (i) 160 concepts overlap with BIOC-GS – i.e., 45% of BIOC-GS and 14% of the learning set; (ii) 138 concepts overlap with HPO-GS – i.e., 30% of HPO-GS and 12% of the learning set. We re-ran Prompt 7 on gpt-4.0 on both corpora and the results – as shown in Table 5 – are encouraging.

**Table 5:** Experimental results on using a random set of concepts for in-context learning

	**Document-level**			**Mention-level**		
	Precision	Recall	F1	Precision	Recall	F1
HPO-GS	0.63	0.29	0.4	0.6	0.19	0.29
BIOC-GS	0.55	0.42	0.48	0.5	0.37	0.43

These results support our assumption that gpt-4.0 would be useful for annotation purposes in a domain-specific setting, without the need to use the entire set of concepts describing the domain to perform in-context learning.

### Concordance across prompts

A complete overview of the pairwise concordance of the outcomes across both models and all prompts is provided in Appendices 2 and 3 in the Supplementary material. We recorded the percentage of common correct and incorrect HPO IDs when considering one model output as base reference. For example, on HPO-GS 51% of the correct HPO IDs extracted by gpt-3.5 Prompt 1 are in common with the correct HPO IDs extracted by gpt-3.5 Prompt 2, with this common set representing 93% of the total correct HPO IDs extracted by the latter.

Overall, the results vary significantly and there is no combination of model—prompt that achieved a high level of agreement on both correctly and incorrectly extracted HPO IDs. A stand-out is perhaps gpt-3.5 Prompt 2 that achieves a rather consistent level of agreement with most of the other prompts on both corpora: (i) on HPO-GS—93% correct in common with Prompt 1—which is expected because Prompt 2 targets conceptually a subset of Prompt 1, 87% with Prompt 7, 84% with gpt-4.0 Prompt 1; (ii) on BIOC-GS – 97% correct in common with Prompt 1, 81% with Prompt 7 and over 80% with all gpt-4.0 experiments except for Prompts 5 and 6.

Appendix 4 in the Supplementary material lists the top 5 incorrectly extracted HPO IDs across all experiments. These HPO IDs are fairly consistent within the context of a model and completely divergent across models. For example, the most common errors of gpt-3.5 are: *Decreased body weight* (HP:0004325), *Intellectual disability, profound* (HP:0002187), *Joint hypermobility* (HP:0001382), *Abnormality of the nervous system* (HP:0000707), while those of gpt-4.0 are: *Poor wound healing* (HP:0001058), *Cerebral hamartoma* (HP:0009731).

It is interesting to note the nature of failures in concept mapping. For example, gpt-3.5 tags the text ‘Angelman’s syndrome’ (a disease not present in HPO) with *Decreased body weight* (HP:0004325 – shown in Fig. 1), and ‘Prader-Willi syndrome’ (another disease not present in HPO), ‘bilateral acoustic neuromas’ or ‘Neurofibromatosis type 2’ with *Intellectual disability, profound* (HP:0002187), while gpt-4.0 tags, consistently, the same text spans, e.g., ‘Angelman’s syndrome’, with *Poor wound healing* (HP:0001058) or ‘Neurofibromatosis type 2’ with *Cerebral hamartoma* (HP:0009731).

### Same model and prompt concordance

A final experiment was performed to understand the concordance across different runs of the same model and prompt. We ran five times the annotation experiment using gpt-4.0 Prompt 1. Overall, all runs achieved the same precision and recall, with very minor differences (± 0.01). The concordance in the results produced by the runs was, however, surprisingly low. Across all runs, we found the common set of: 75.82% of all correctly identified HPO IDs; 28.09% of all incorrectly identified HPO IDs, and 86.6% of all concepts not found by the models. This shows a high level of divergence in concept mapping errors produced by the individual runs.

## Limitations

A summary of the limitations derived from the experiments discussed above is listed below:

- The in-context learning strategy adopted in our experiments, while surpassing the state of the art in some cases, differs from the task of open phenotype concept recognition. Due to limitations in the size of the input data, we restricted the examples to only the concepts present in the gold standard. In a real-world scenario, this set of concepts is unknown. However, a retrieval augmented generation (RAG) approach would be feasible to provide the model with the most relevant HPO terms as context. Further, our experiments did, however, show that ontology stratification strategies could be employed as an alternative to using the entire ontology – e.g., domain-specific selection. Cost is still a prohibitive factor for this approach. For example, the in-context learning experiments on BIOC-GS costed USD $50, which used only 454 entries with an average length of 56 characters + the learning component of 358 unique ontology concepts (~ 12KB)
- The performance of the model is non-deterministic. Executing the same prompt over the same input leads to slightly different results. This is particularly challenging as it hinders the establishment of an accurate ground truth and leaves a degree of uncertainty in completeness always associated with the outcomes.
- The choice of wording in the prompt influences the results. While this is expected (hence the need for iterative prompt engineering), it is also remarkably challenging when considering the lack of concordance between the outcomes—as shown in Appendices 2 and 3 in the Supplementary material (e.g., prompts that have been iterated on produce HPO IDs that are not found by subsequent prompts)

Our study has its limitations as well. Firstly, the variety of prompts included in the experiments is limited. As discussed in the experimental setup, we covered low-barrier prompts, showcasing the trade-off between approaches at the lower end of the cost scale and the context in which they can produce reasonable results. Secondly, LLMs are trained using publicly available data, which could include the HPO_GS, since it was initially published in 2015 and in turn could lead to a test data leakage. While this is indeed a realistic scenario, the expectations associated with it do not seem to be met by the poor results listed in Table 1 for Prompts 1–6 (we exclude Prompt 7 since it addresses directly in-context learning). Moreover, the same scenario does not hold for BIOC-GS (since it was not publicly available at the time of our experiments) and yet the results are significantly better. We argue that test data leakage is unlikely for scenarios similar to the ones presented in this paper because of the boost in efficiency provided by in-context learning. The F1 scores achieved on Prompt 7 are significantly better than all others, and hence we argue that the gap between results would have been smaller if the LLM would have just memorized the answers to the task (and hence the examples provided in Prompt 7 would not have helped as much).

## Conclusion

This paper presents a study that assesses the capabilities of the GPT models underpinning ChatGPT to perform phenotype concept recognition, using concepts grounded in the Human Phenotype Ontology, assuming a need for manual curation / annotation of publications or clinical records. The experimental setup covered both gpt-3.5 and gpt-4.0 and a series of seven prompts ranging from direct instructions to perform the task by name to chains of named entity recognition followed by concept mapping and to in-context learning. LLMs learn ontologies imperfectly. In-context approaches get around imperfect training. Providing entire ontologies in-context produces the desired outcomes in principle but is impractical. Ontologies are frequently bigger than the context windows supported by current LLMs, hence needing a priori knowledge of the target area of the ontology to be pre-filtered for the desired terms. However, a retrieval augmented generation (RAG) approach might be feasible in future work to provide a benefit similar to providing the entire ontology. Our results show that using in-context learning with the pre-filtered terms leads to these models surpassing the best-in-class tools, which are either using BERT-based architectures or more classical natural language processing pipelines.

The main challenges to a direct adoption of these models, document by our error analysis include the non-deterministic outputs of the models, the lack of concordance between different prompt outputs, as well as between different runs with the same prompt. Unlike other use cases, hallucinations do not affect the task we have focused on.

### Supplementary Information

**Additional file 1: Appendix 1.** Prompt 7 example (PMID: 292745). **Appendix 2.** Pairwise concordance across all models and prompts using the HPO-GS corpus. **Appendix 3.** Pairwise concordance across all models and prompts using the BIOC-GS corpus. **Appendix 4.** Top 5 incorrectly extracted HPO IDs across all experiments.

## Data Availability

The code used to annotate the corpora and perform the evaluation is available at: https://github.com/tudorgroza/code-for-papers. HPO-GS is available in the same repository. BIOC-GS requires registration to BioCreative VIII (https://biocreative.bioinformatics.udel.edu/news/biocreative-viii/) or can be requested from the organisers of Track 3: Genetic Phenotype Extraction and Normalization from Dysmorphology Physical Examination Entries (genetic conditions in pediatric patients).

## References

[1] Taruscio D, Groft SC, Cederroth H (2015). Undiagnosed Diseases Network International (UDNI): White paper for global actions to meet patient needs. Mol Genet Metab.

[2] Boycott KM, Azzariti DR, Hamosh A, Rehm HL (2022). Seven years since the launch of the Matchmaker Exchange: The evolution of genomic matchmaking. Hum Mutat.

[3] Jacobsen JOB, Baudis M, Baynam GS (2022). The GA4GH Phenopacket schema defines a computable representation of clinical data. Nat Biotechnol.

[4] Smedley D, Schubach M, Jacobsen JOB (2016). A whole-genome analysis framework for effective identification of pathogenic regulatory variants in Mendelian disease. Am J Hum Genet.

[5] Son JH, Xie G, Yuan C (2018). Deep Phenotyping on electronic health records facilitates genetic diagnosis by clinical exomes. Am J Hum Genet.

[6] Clark MM, Stark Z, Farnaes L (2018). Meta-analysis of the diagnostic and clinical utility of genome and exome sequencing and chromosomal microarray in children with suspected genetic diseases. NPJ Genom Med.

[7] Robinson PN, Köhler S, Bauer S (2008). The Human Phenotype Ontology: a tool for annotating and analyzing human hereditary disease. Am J Hum Genet.

[8] Köhler S, Carmody L, Vasilevsky N (2019). Expansion of the Human Phenotype Ontology (HPO) knowledge base and resources. Nucleic Acids Res.

[9] Shefchek KA, Harris NL, Gargano M (2020). The Monarch Initiative in 2019: an integrative data and analytic platform connecting phenotypes to genotypes across species. Nucleic Acids Res.

[10] 10,000 Genomes Project Pilot Investigators (2021). 100,000 genomes pilot on rare-disease diagnosis in health care - preliminary report. N Engl J Med.

[11] Arbabi A, Adams DR, Fidler S, Brudno M (2019). Identifying clinical terms in medical text using ontology-guided machine learning. JMIR Med Inform.

[12] Luo L, Yan S, Lai P-T (2021). PhenoTagger: a hybrid method for phenotype concept recognition using human phenotype ontology. Bioinformatics.

[13] Krishnan R, Rajpurkar P, Topol EJ (2022). Self-supervised learning in medicine and healthcare. Nat Biomed Eng.

[14] Thirunavukarasu AJ (2023). Large language models in medicine. Nat Med.

[15] Lee J (2020). BioBERT: a pre-trained biomedical language representation model for biomedical text mining. Bioinformatics.

[16] Moor M (2023). Foundation models for generalist medical artificial intelligence. Nature.

[17] Yu G, Tinn R, Cheng H (2021). Domain-specific language model pretraining for biomedical natural language processing. ACM Trans Comp Healthc (HEALTH).

[18] Luo R, Sun L, Xia Y, Qin T, Zhang S, Poon H, Liu TY (2022). BioGPT: generative pre-trained transformer for biomedical text generation and mining. Brief Bioinform.

[19] Ding B, Qin C, Liu L, Chia YK, Joty S, Li B, Bing L. Is GPT-3 a Good Data Annotator? Proceedings of the 61st Annual Meeting of the Association for Computational Linguistics. 2023;1:11173–11195. LongPapers.

[20] Gray M, Savelka J, Oliver W, Ashley K (2023). Can GPT Alleviate the Burden of Annotation? Proceedings of JURIX 2023 36th International Conference on Legal Knowledge and Information Systems.

[21] Chen Q, Du J, Hu Y, Keloth VK, Peng X, Raja K, Zhang R, Lu X, Xu H. Large language models in biomedical natural language processing: benchmarks, baselines, and recommendations. 2023. arXiv preprint, arXiv:2305.16326.

[22] Groza T, Köhler S, Doelken S, Collier N, Oellrich A, Smedley D, Couto FM, Baynam G, Zankl A, Robinson PN (2015). Automatic concept recognition using the human phenotype ontology reference and test suite corpora. Database (Oxford).

[23] Lobo M, Lamurias A, Couto FM (2017). Identifying Human phenotype terms by combining machine learning and validation rules. Biomed Res Inte.

[24] Weissenbacher D, Rawal S, Zhao X, Priestley JRC, Szigety KM, Schmidt SF, Higgins MJ, Magge A, O’Connor K, Gonzalez-Hernandez G, Campbell IM. PheNorm, a language model normalizer of physical examinations from genetics clinical notes. medRxiv 2023.10.16.23296894. 10.1101/2023.10.16.23296894.

[25] Liu C, Kury FSP, Li Z, Ta C, Wang K, Weng C (2019). Doc2Hpo: a web application for efficient and accurate HPO concept curation. Nucleic Acids Res.

[26] Deisseroth CA, Birgmeier J, Bodle EE (2019). ClinPhen extracts and prioritizes patient phenotypes directly from medical records to expedite genetic disease diagnosis. Genet Med.

[27] Jonquet C, Shah NH, Musen MA (2009). The Open Biomedical Annotator. AMIA Joint Summit Transl Bioinform.

