# Supplementary material for: An evaluation of GPT models for phenotype concept recognition
Source: BMC Med Inform Decis Mak. 2024 Jan 31;24:30. doi: 10.1186/s12911-024-02439-w (PMC10829255; doi:10.1186/s12911-024-02439-w)
Supplement: Supplementary file 1 — Additional file 1: Appendix 1. Prompt 7 example (PMID: 292745). Appendix 2. Pairwise concordance across all models and prompts using the HPO-GS corpus. Appendix 3. Pairwise concordance across all models and prompts using the BIOC-GS corpus. Appendix 4. Top 5 incorrectly extracted HPO IDs across all experiments. [file 12911_2024_2439_MOESM1_ESM.docx]

**APPENDIX 1: Prompt 7 example (PMID: 292745)**

Examples: The Human Phenotype Ontology defines phenotype concepts using the following label – HPO ID associations:

Hypospadias // HP:0000047

Ureteral duplication // HP:0000073

Abnormality of the kidney // HP:0000077

Abnormality of the genital system // HP:0000078

Abnormality of the urinary system // HP:0000079

Duplicated collecting system // HP:0000081

Ectopic kidney // HP:0000086

Renal hypoplasia // HP:0000089

Renal agenesis // HP:0000104

Renal dysplasia // HP:0000110

Polycystic kidney dysplasia // HP:0000113

Unilateral renal agenesis // HP:0000122

Abnormality of the ovary // HP:0000137

Rectovaginal fistula // HP:0000143

Abnormality of the mouth // HP:0000153

Wide mouth // HP:0000154

Abnormal lip morphology // HP:0000159

Abnormality of the dentition // HP:0000164

Abnormality of the gingiva // HP:0000168

Microglossia // HP:0000171

Abnormal palate morphology // HP:0000174

Cleft palate // HP:0000175

Abnormal upper lip morphology // HP:0000177

Movement abnormality of the tongue // HP:0000182

Lower lip pit // HP:0000196

Orofacial cleft // HP:0000202

Trismus // HP:0000211

Hydrocephalus // HP:0000238

Parietal bossing // HP:0000242

Brachycephaly // HP:0000248

Microcephaly // HP:0000252

Macrocephaly // HP:0000256

Abnormality of the face // HP:0000271

Malar flattening // HP:0000272

Abnormal mandible morphology // HP:0000277

Coarse facial features // HP:0000280

Epicanthus // HP:0000286

Abnormality of the philtrum // HP:0000288

Mandibular prognathia // HP:0000303

Pointed chin // HP:0000307

Microretrognathia // HP:0000308

Hypertelorism // HP:0000316

Short philtrum // HP:0000322

Micrognathia // HP:0000347

Strabismus // HP:0000486

Deeply set eye // HP:0000490

Downslanted palpebral fissures // HP:0000494

Abnormality of eye movement // HP:0000496

Ptosis // HP:0000508

Cataract // HP:0000518

Subcapsular cataract // HP:0000523

Abnormality iris morphology // HP:0000525

Myopia // HP:0000545

Keratoconus // HP:0000563

Microphthalmia // HP:0000568

Exotropia // HP:0000577

Nasolacrimal duct obstruction // HP:0000579

Coloboma // HP:0000589

Blindness // HP:0000618

Nystagmus // HP:0000639

Widely spaced teeth // HP:0000687

Atypical behavior // HP:0000708

Autism // HP:0000717

Aggressive behavior // HP:0000718

Autistic behavior // HP:0000729

Inappropriate laughter // HP:0000748

Paroxysmal bursts of laughter // HP:0000749

Hyperactivity // HP:0000752

Abnormal peripheral nervous system morphology // HP:0000759

Peripheral axonal degeneration // HP:0000764

Abnormal sternum morphology // HP:0000766

Pectus excavatum // HP:0000767

Abnormal rib morphology // HP:0000772

Hyperparathyroidism // HP:0000843

Abnormality of the outer ear // HP:0000356

Abnormality of the inner ear // HP:0000359

Tinnitus // HP:0000360

Hearing abnormality // HP:0000364

Hearing impairment // HP:0000365

Abnormality of the nose // HP:0000366

Low-set ears // HP:0000369

Abnormality of the middle ear // HP:0000370

Abnormal cochlea morphology // HP:0000375

Incomplete partition of the cochlea type II // HP:0000376

Abnormal pinna morphology // HP:0000377

Cupped ear // HP:0000378

Stapes ankylosis // HP:0000381

Abnormal periauricular region morphology // HP:0000383

Preauricular skin tag // HP:0000384

Lop ear // HP:0000394

Recurrent otitis media // HP:0000403

Conductive hearing impairment // HP:0000405

Sensorineural hearing impairment // HP:0000407

Mixed hearing impairment // HP:0000410

Atresia of the external auditory canal // HP:0000413

Wide nasal bridge // HP:0000431

Depressed nasal tip // HP:0000437

Abnormality of the neck // HP:0000464

Abnormality of the skin // HP:0000951

Hyperpigmentation of the skin // HP:0000953

Cafe-au-lait spot // HP:0000957

Edema // HP:0000969

Hypopigmentation of the skin // HP:0001010

Multiple lipomas // HP:0001012

Albinism // HP:0001022

Numerous nevi // HP:0001054

Milia // HP:0001056

Pterygium // HP:0001059

Neurofibroma // HP:0001067

Ocular albinism // HP:0001107

Limbal dermoid // HP:0001140

Orbital cyst // HP:0001144

Abnormality of the hand // HP:0001155

Brachydactyly // HP:0001156

Syndactyly // HP:0001159

Arachnodactyly // HP:0001166

Abnormal thumb morphology // HP:0001172

Preaxial hand polydactyly // HP:0001177

Hand clenching // HP:0001188

Ulnar deviation of the hand or of fingers of the hand // HP:0001193

Triphalangeal thumb // HP:0001199

Distal symphalangism of hands // HP:0001204

Failure to thrive // HP:0001508

Growth delay // HP:0001510

Intrauterine growth retardation // HP:0001511

Obesity // HP:0001513

Anteriorly placed anus // HP:0001545

Overgrowth // HP:0001548

Oligohydramnios // HP:0001562

Abnormality of the nail // HP:0001597

Hypernasal speech // HP:0001611

Premature birth // HP:0001622

Abnormal heart morphology // HP:0001627

Truncus arteriosus // HP:0001660

Bradycardia // HP:0001662

Abnormal foot morphology // HP:0001760

Talipes equinovarus // HP:0001762

Toe syndactyly // HP:0001770

Bilateral talipes equinovarus // HP:0001776

Small nail // HP:0001792

Anonychia // HP:0001798

Rocker bottom foot // HP:0001838

Talipes // HP:0001883

Abnormal clavicle morphology // HP:0000889

Cervical ribs // HP:0000891

Bifid ribs // HP:0000892

Sprengel anomaly // HP:0000912

Abnormality of the skeletal system // HP:0000924

Abnormality of the vertebral column // HP:0000925

Abnormal facial shape // HP:0001999

Frontal bossing // HP:0002007

Potter facies // HP:0002009

Abnormality of the abdominal organs // HP:0002012

Vomiting // HP:0002013

Dysphagia // HP:0002015

Anal atresia // HP:0002023

Abdominal pain // HP:0002027

Abnormal rectum morphology // HP:0002034

Cerebral atrophy // HP:0002059

Abnormal cerebral morphology // HP:0002060

Gait ataxia // HP:0002066

Bilateral tonic-clonic seizure // HP:0002069

Limb ataxia // HP:0002070

Abnormal lung morphology // HP:0002088

Pulmonary hypoplasia // HP:0002089

Pneumothorax // HP:0002107

Abnormal cerebral ventricle morphology // HP:0002118

Generalized myoclonic seizure // HP:0002123

Status epilepticus // HP:0002133

Broad-based gait // HP:0002136

Abnormality of speech or vocalization // HP:0002167

Intellectual disability, profound // HP:0002187

Dysgenesis of the cerebellar vermis // HP:0002195

White forelock // HP:0002211

Premature graying of hair // HP:0002216

Poor motor coordination // HP:0002275

Global brain atrophy // HP:0002283

Incoordination // HP:0002311

Vertigo // HP:0002321

Drowsiness // HP:0002329

Delayed ossification of carpal bones // HP:0001216

Intellectual disability // HP:0001249

Seizure // HP:0001250

Ataxia // HP:0001251

Hypotonia // HP:0001252

Intellectual disability, mild // HP:0001256

Spasticity // HP:0001257

Global developmental delay // HP:0001263

Hemiparesis // HP:0001269

Motor delay // HP:0001270

Cerebellar atrophy // HP:0001272

Gait disturbance // HP:0001288

Abnormal cranial nerve morphology // HP:0001291

Encephalopathy // HP:0001298

Abnormal cerebellum morphology // HP:0001317

Neonatal hypotonia // HP:0001319

Muscle weakness // HP:0001324

Specific learning disability // HP:0001328

Myoclonus // HP:0001336

Absent speech // HP:0001344

Hyperreflexia // HP:0001347

Craniosynostosis // HP:0001363

Abnormal joint morphology // HP:0001367

Flexion contracture // HP:0001371

Hip dysplasia // HP:0001385

Cerebral calcification // HP:0002514

Increased intracranial pressure // HP:0002516

Inability to walk // HP:0002540

Episodic abdominal pain // HP:0002574

Colitis // HP:0002583

Polyphagia // HP:0002591

Hypotension // HP:0002615

Madelung deformity // HP:0003067

Defective DNA repair after ultraviolet radiation damage // HP:0003079

Limb joint contracture // HP:0003121

Skeletal muscle atrophy // HP:0003202

Prominent scrotal raphe // HP:0003246

Spina bifida occulta // HP:0003298

Spondylolisthesis // HP:0003302

Spondylolysis // HP:0003304

Spinal canal stenosis // HP:0003416

Abnormal 2nd finger morphology // HP:0004100

Short middle phalanx of the 5th finger // HP:0004220

Abnormal gastric mucosa morphology // HP:0004295

Abnormal renal collecting system morphology // HP:0004742

Rectoperineal fistula // HP:0004792

Absence of the pulmonary valve // HP:0005134

Internal carotid artery hypoplasia // HP:0005290

Bridged sella turcica // HP:0005449

EEG abnormality // HP:0002353

Sleep disturbance // HP:0002360

Abnormal brainstem morphology // HP:0002363

Spina bifida // HP:0002414

Language impairment // HP:0002463

Hyperkinetic movements // HP:0002487

Absent trapezoid bone // HP:0006106

Abnormal finger flexion crease // HP:0006143

Proximal symphalangism of hands // HP:0006152

Calcification of falx cerebri // HP:0005462

Fusion of middle ear ossicles // HP:0005473

Secondary microcephaly // HP:0005484

Duplication of renal pelvis // HP:0005580

Hypopigmentation of hair // HP:0005599

Positional foot deformity // HP:0005656

Distal arthrogryposis // HP:0005684

Short middle phalanx of finger // HP:0005819

Biliary atresia // HP:0005912

Multiple pulmonary cysts // HP:0005948

Paraspinal neurofibroma // HP:0006751

Dysplastic patella // HP:0006446

Proximal femoral epiphysiolysis // HP:0006461

Abnormality of the alveolar ridges // HP:0006477

Cerebellar calcifications // HP:0007352

Focal-onset seizure // HP:0007359

Confetti-like hypopigmented macules // HP:0007449

Generalized hypopigmentation // HP:0007513

Bilateral microphthalmos // HP:0007633

Lacrimal duct stenosis // HP:0007678

Vitreoretinopathy // HP:0007773

Posterior subcapsular cataract // HP:0007787

Scoliosis // HP:0002650

Skeletal dysplasia // HP:0002652

Basal cell carcinoma // HP:0002671

Large foramen magnum // HP:0002700

Bilateral cleft lip and palate // HP:0002744

Congenital contracture // HP:0002803

Kyphosis // HP:0002808

Abnormality of limb bone morphology // HP:0002813

Abnormality of the lower limb // HP:0002814

Abnormality of the upper limb // HP:0002817

Abnormal morphology of the radius // HP:0002818

Multiple joint contractures // HP:0002828

Meningioma // HP:0002858

Medulloblastoma // HP:0002885

Ependymoma // HP:0002888

Hemivertebrae // HP:0002937

Vertebral fusion // HP:0002948

Lacrimal duct aplasia // HP:0007925

Malformed lacrimal duct // HP:0007993

Peripheral opacification of the cornea // HP:0008011

Neoplasm of the skin // HP:0008069

Ankylosis of feet small joints // HP:0008090

Intervertebral disc degeneration // HP:0008419

Cochlear malformation // HP:0008554

Hypoplasia of the cochlea // HP:0008586

Supraauricular pit // HP:0008606

Bilateral sensorineural hearing impairment // HP:0008619

Nonprogressive encephalopathy // HP:0007030

Atypical absence seizure // HP:0007270

Unilateral vestibular schwannoma // HP:0009590

Abnormality of the vestibulocochlear nerve // HP:0009591

Astrocytoma // HP:0009592

Peripheral schwannoma // HP:0009593

Retinal hamartoma // HP:0009594

Carpal synostosis // HP:0009702

Adenoma sebaceum // HP:0009720

Rhabdomyoma // HP:0009730

Glioma // HP:0009733

Spinal neurofibroma // HP:0009735

Lisch nodules // HP:0009737

Ankyloblepharon // HP:0009755

Popliteal pterygium // HP:0009756

Short thumb // HP:0009778

Branchial anomaly // HP:0009794

Branchial fistula // HP:0009795

Branchial cyst // HP:0009796

Cholesteatoma // HP:0009797

Peripheral neuropathy // HP:0009830

Mononeuropathy // HP:0009831

Aplasia/Hypoplasia of the middle phalanges of the hand // HP:0009843

Aplasia of the distal phalanges of the hand // HP:0009881

Abnormal temporal bone morphology // HP:0009911

Duane anomaly // HP:0009921

Partial duplication of thumb phalanx // HP:0009944

Type A brachydactyly // HP:0009370

Type A1 brachydactyly // HP:0009371

Type A2 brachydactyly // HP:0009372

Type C brachydactyly // HP:0009373

Fever // HP:0001945

Abnormal scalp morphology // HP:0001965

Short 4th metacarpal // HP:0010044

Short 5th metacarpal // HP:0010047

Short hallux // HP:0010109

Pseudoepiphyses of the phalanges of the hand // HP:0010235

Ulnar deviation of the 2nd finger // HP:0009464

Ulnar deviation of finger // HP:0009465

Pseudoepiphysis of the 2nd finger // HP:0009495

Short 2nd finger // HP:0009536

Vestibular schwannoma // HP:0009588

Bilateral vestibular schwannoma // HP:0009589

Polydactyly // HP:0010442

Long toe // HP:0010511

Coronal hypospadias // HP:0008743

Feeding difficulties in infancy // HP:0008872

Postnatal growth retardation // HP:0008897

Generalized neonatal hypotonia // HP:0008935

Abnormal axial skeleton morphology // HP:0009121

Protruding tongue // HP:0010808

Atonic seizure // HP:0010819

Mild global developmental delay // HP:0011342

Moderate global developmental delay // HP:0011343

Localized skin lesion // HP:0011355

Morphological abnormality of the semicircular canal // HP:0011380

Abnormality of the incus // HP:0011453

Feeding difficulties // HP:0011968

Camptodactyly // HP:0012385

Pain // HP:0012531

Bilateral renal dysplasia // HP:0012582

Involuntary movements // HP:0004305

Ventricular arrhythmia // HP:0004308

Short stature // HP:0004322

Abnormality of bone mineral density // HP:0004348

Abnormal circulating calcium concentration // HP:0004363

Neoplasm of the nervous system // HP:0004375

Abnormality of the anus // HP:0004378

Abnormality of the middle ear ossicles // HP:0004452

Dilatated internal auditory canal // HP:0004458

Postauricular pit // HP:0004464

Preauricular pit // HP:0004467

Relative macrocephaly // HP:0004482

Clinodactyly // HP:0030084

Spinal cord tumor // HP:0010302

Profound global developmental delay // HP:0012736

Papilloma // HP:0012740

Neurodevelopmental abnormality // HP:0012759

Overlapping fingers // HP:0010557

Odontogenic keratocysts of the jaw // HP:0010603

Chalazion // HP:0010605

Skin tags // HP:0010609

Palmar pits // HP:0010610

Plantar pits // HP:0010612

Angiofibromas // HP:0010615

Cardiac fibroma // HP:0010617

Ovarian fibroma // HP:0010618

Ectopic calcification // HP:0010766

Erythema // HP:0010783

Happy demeanor // HP:0040082

Premature skin wrinkling // HP:0100678

Abnormality of the seventh cranial nerve // HP:0010827

EEG with persistent abnormal rhythmic activity // HP:0010846

EEG with spike-wave complexes (2.5-3.5 Hz) // HP:0010848

Intellectual disability, severe // HP:0010864

Bilateral renal agenesis // HP:0010958

Epileptic spasm // HP:0011097

Papule // HP:0200034

Epidermoid cyst // HP:0200040

Malignant mesothelioma // HP:0100001

Neoplasm of the central nervous system // HP:0100006

Neoplasm of the peripheral nervous system // HP:0100007

Schwannoma // HP:0100008

Intracranial meningioma // HP:0100009

Spinal meningioma // HP:0100010

Scleral schwannoma // HP:0100011

Epiretinal membrane // HP:0100014

Capsular cataract // HP:0100017

Cortical cataract // HP:0100019

Posterior capsular cataract // HP:0100020

Cerebral palsy // HP:0100021

Abnormality of movement // HP:0100022

Recurrent hand flapping // HP:0100023

Conspicuously happy disposition // HP:0100024

Sarcoma // HP:0100242

Fibrosarcoma // HP:0100244

Ectrodactyly // HP:0100257

Preaxial polydactyly // HP:0100258

Proximal symphalangism // HP:0100264

Paramedian lip pit // HP:0100269

Branchial sinus // HP:0100272

Gustatory lacrimation // HP:0100274

Ulcerative colitis // HP:0100279

Unilateral cleft lip // HP:0100333

Unilateral cleft palate // HP:0100334

Bilateral cleft lip // HP:0100336

Neoplasm of the endocrine system // HP:0100568

Short ear // HP:0400005

Task: Using the list above, find Human Phenotype Ontology concepts in the following text and return their associated IDs for every appearance in the text: This paper is based on our experience with the Gorlin-Goltz syndrome and on data from 14 patients of the Nordwestdeutsche Kieferklinik in whom this disorder was detected, treated and followed up. A clinical concept has been produced, with a diagnostic check list including a genetic and a dermatological routine work up as well as a radiological survey of the jaws and skeleton. Whenever multiple basal cell carcinomas plus the typical jaw lesions are found in a patient, the diagnosis is easy. A minimum diagnostic criterion is the combination of either the skin tumours or multiple odontogenic keratocysts plus a positive family history for this disorder, bifid ribs, lamellar calcification of the falx cerebri or any one of the skeletal abnormalities typical of this syndrome. All those in whom this disorder is diagnosed or suspected should be followed up for the rest of their lives. The family should be examined and genetic counselling should be offered.

**APPENDIX 2: Pairwise concordance across all models and prompts using the HPO-GS corpus**

**Base:** gpt-3.5, Prompt 1

| **Model** | **Prompt** | **Common correct in base prompt** | **Common correct in comparing prompt** | **Common incorrect in base prompt** | **Common incorrect in comparing prompt** |
| --- | --- | --- | --- | --- | --- |
| 3.5 | 2 | 0.51 | 0.93 | 0.27 | 0.59 |
|  | 3 | 0.57 | 0.51 | 0.21 | 0.03 |
|  | 5 | 0.25 | 0.59 | 0.1 | 0.05 |
|  | 6 | 0.37 | 0.63 | 0.12 | 0.1 |
|  | 4 | 0.62 | 0.5 | 0.29 | 0.04 |
|  | 7 | 0.79 | 0.43 | 0.14 | 0.06 |
| 4.0 | 1 | 0.77 | 0.5 | 0.09 | 0.04 |
|  | 2 | 0.77 | 0.52 | 0.09 | 0.05 |
|  | 3 | 0.72 | 0.53 | 0.08 | 0.04 |
|  | 5 | 0.5 | 0.51 | 0.06 | 0.03 |
|  | 6 | 0.38 | 0.52 | 0.04 | 0.03 |
|  | 4 | 0.77 | 0.46 | 0.1 | 0.03 |
|  | 7 | 0.8 | 0.4 | 0.09 | 0.02 |

**Base:** gpt-3.5, Prompt 2

| **Model** | **Prompt** | **Common correct in base prompt** | **Common correct in comparing prompt** | **Common incorrect in base prompt** | **Common incorrect in comparing prompt** |
| --- | --- | --- | --- | --- | --- |
| 3.5 | 1 | 0.93 | 0.51 | 0.59 | 0.27 |
|  | 3 | 0.64 | 0.31 | 0.18 | 0.01 |
|  | 5 | 0.25 | 0.33 | 0.08 | 0.02 |
|  | 6 | 0.44 | 0.42 | 0.12 | 0.05 |
|  | 4 | 0.65 | 0.29 | 0.34 | 0.02 |
|  | 7 | 0.87 | 0.26 | 0.2 | 0.04 |
| 4.0 | 1 | 0.84 | 0.3 | 0.13 | 0.03 |
|  | 2 | 0.85 | 0.31 | 0.13 | 0.03 |
|  | 3 | 0.82 | 0.33 | 0.13 | 0.03 |
|  | 5 | 0.59 | 0.33 | 0.1 | 0.02 |
|  | 6 | 0.49 | 0.36 | 0.05 | 0.02 |
|  | 4 | 0.86 | 0.28 | 0.14 | 0.02 |
|  | 7 | 0.85 | 0.24 | 0.13 | 0.01 |

**Base:** gpt-3.5, Prompt 3

| **Model** | **Prompt** | **Common correct in base prompt** | **Common correct in comparing prompt** | **Common incorrect in base prompt** | **Common incorrect in comparing prompt** |
| --- | --- | --- | --- | --- | --- |
| 3.5 | 1 | 0.51 | 0.57 | 0.03 | 0.21 |
|  | 2 | 0.31 | 0.64 | 0.01 | 0.18 |
|  | 5 | 0.22 | 0.57 | 0.02 | 0.06 |
|  | 6 | 0.32 | 0.61 | 0.03 | 0.16 |
|  | 4 | 0.63 | 0.57 | 0.13 | 0.11 |
|  | 7 | 0.69 | 0.42 | 0.04 | 0.1 |
| 4.0 | 1 | 0.65 | 0.48 | 0.01 | 0.04 |
|  | 2 | 0.65 | 0.48 | 0.01 | 0.05 |
|  | 3 | 0.59 | 0.48 | 0.01 | 0.05 |
|  | 5 | 0.39 | 0.45 | 0.01 | 0.03 |
|  | 6 | 0.32 | 0.48 | 0.01 | 0.03 |
|  | 4 | 0.65 | 0.43 | 0.02 | 0.04 |
|  | 7 | 0.65 | 0.36 | 0.02 | 0.03 |

**Base:** gpt-3.5, Prompt 4

| **Model** | **Prompt** | **Common correct in base prompt** | **Common correct in comparing prompt** | **Common incorrect in base prompt** | **Common incorrect in comparing prompt** |
| --- | --- | --- | --- | --- | --- |
| 3.5 | 1 | 0.5 | 0.62 | 0.04 | 0.29 |
|  | 2 | 0.29 | 0.65 | 0.02 | 0.34 |
|  | 3 | 0.57 | 0.63 | 0.11 | 0.13 |
|  | 5 | 0.21 | 0.61 | 0.02 | 0.07 |
|  | 6 | 0.29 | 0.62 | 0.02 | 0.16 |
|  | 7 | 0.68 | 0.46 | 0.04 | 0.12 |
| 4.0 | 1 | 0.63 | 0.51 | 0.01 | 0.05 |
|  | 2 | 0.62 | 0.51 | 0.01 | 0.05 |
|  | 3 | 0.56 | 0.51 | 0.02 | 0.06 |
|  | 5 | 0.41 | 0.52 | 0.01 | 0.02 |
|  | 6 | 0.3 | 0.5 | 0.01 | 0.03 |
|  | 4 | 0.63 | 0.47 | 0.01 | 0.03 |
|  | 7 | 0.65 | 0.41 | 0.03 | 0.04 |

**Base:** gpt-3.5, Prompt 5

| **Model** | **Prompt** | **Common correct in base prompt** | **Common correct in comparing prompt** | **Common incorrect in base prompt** | **Common incorrect in comparing prompt** |
| --- | --- | --- | --- | --- | --- |
| 3.5 | 1 | 0.59 | 0.25 | 0.05 | 0.1 |
|  | 2 | 0.33 | 0.25 | 0.02 | 0.08 |
|  | 3 | 0.57 | 0.22 | 0.06 | 0.02 |
|  | 6 | 0.54 | 0.4 | 0.08 | 0.14 |
|  | 4 | 0.61 | 0.21 | 0.07 | 0.02 |
|  | 7 | 0.71 | 0.17 | 0.03 | 0.03 |
| 4.0 | 1 | 0.64 | 0.18 | 0.02 | 0.02 |
|  | 2 | 0.63 | 0.18 | 0.02 | 0.02 |
|  | 3 | 0.58 | 0.18 | 0.02 | 0.02 |
|  | 5 | 0.42 | 0.18 | 0.01 | 0.01 |
|  | 6 | 0.34 | 0.19 | 0 | 0.01 |
|  | 4 | 0.67 | 0.17 | 0.02 | 0.01 |
|  | 7 | 0.68 | 0.15 | 0.01 | 0 |

**Base:** gpt-3.5, Prompt 6

| **Model** | **Prompt** | **Common correct in base prompt** | **Common correct in comparing prompt** | **Common incorrect in base prompt** | **Common incorrect in comparing prompt** |
| --- | --- | --- | --- | --- | --- |
| 3.5 | 1 | 0.63 | 0.37 | 0.1 | 0.12 |
|  | 2 | 0.42 | 0.44 | 0.05 | 0.12 |
|  | 3 | 0.61 | 0.32 | 0.16 | 0.03 |
|  | 5 | 0.4 | 0.54 | 0.14 | 0.08 |
|  | 4 | 0.62 | 0.29 | 0.16 | 0.02 |
|  | 7 | 0.74 | 0.23 | 0.08 | 0.04 |
| 4.0 | 1 | 0.73 | 0.28 | 0.04 | 0.02 |
|  | 2 | 0.72 | 0.28 | 0.04 | 0.02 |
|  | 3 | 0.68 | 0.29 | 0.04 | 0.02 |
|  | 5 | 0.46 | 0.27 | 0.03 | 0.02 |
|  | 6 | 0.38 | 0.3 | 0.01 | 0.01 |
|  | 4 | 0.74 | 0.25 | 0.03 | 0.01 |
|  | 7 | 0.68 | 0.2 | 0.03 | 0.01 |

**Base:** gpt-3.5, Prompt 7

| **Model** | **Prompt** | **Common correct in base prompt** | **Common correct in comparing prompt** | **Common incorrect in base prompt** | **Common incorrect in comparing prompt** |
| --- | --- | --- | --- | --- | --- |
| 3.5 | 1 | 0.43 | 0.79 | 0.06 | 0.14 |
|  | 2 | 0.26 | 0.87 | 0.04 | 0.2 |
|  | 3 | 0.42 | 0.69 | 0.1 | 0.04 |
|  | 5 | 0.17 | 0.71 | 0.03 | 0.03 |
|  | 6 | 0.23 | 0.74 | 0.04 | 0.08 |
|  | 4 | 0.46 | 0.68 | 0.12 | 0.04 |
| 4.0 | 1 | 0.57 | 0.68 | 0.03 | 0.04 |
|  | 2 | 0.56 | 0.69 | 0.03 | 0.04 |
|  | 3 | 0.52 | 0.7 | 0.03 | 0.04 |
|  | 5 | 0.36 | 0.67 | 0.02 | 0.03 |
|  | 6 | 0.27 | 0.67 | 0.01 | 0.02 |
|  | 4 | 0.58 | 0.63 | 0.04 | 0.03 |
|  | 7 | 0.7 | 0.65 | 0.05 | 0.02 |

**Base:** gpt-4.0, Prompt 1

| **Model** | **Prompt** | **Common correct in base prompt** | **Common correct in comparing prompt** | **Common incorrect in base prompt** | **Common incorrect in comparing prompt** |
| --- | --- | --- | --- | --- | --- |
| 3.5 | 1 | 0.5 | 0.77 | 0.04 | 0.09 |
|  | 2 | 0.3 | 0.84 | 0.03 | 0.13 |
|  | 3 | 0.48 | 0.65 | 0.04 | 0.01 |
|  | 5 | 0.18 | 0.64 | 0.02 | 0.02 |
|  | 6 | 0.28 | 0.73 | 0.02 | 0.04 |
|  | 4 | 0.51 | 0.63 | 0.05 | 0.01 |
|  | 7 | 0.68 | 0.57 | 0.04 | 0.03 |
| 4.0 | 2 | 0.91 | 0.93 | 0.63 | 0.65 |
|  | 3 | 0.75 | 0.84 | 0.33 | 0.33 |
|  | 5 | 0.51 | 0.8 | 0.23 | 0.24 |
|  | 6 | 0.37 | 0.76 | 0.18 | 0.28 |
|  | 4 | 0.83 | 0.75 | 0.4 | 0.27 |
|  | 7 | 0.74 | 0.57 | 0.13 | 0.05 |

**Base:** gpt-4.0, Prompt 2

| **Model** | **Prompt** | **Common correct in base prompt** | **Common correct in comparing prompt** | **Common incorrect in base prompt** | **Common incorrect in comparing prompt** |
| --- | --- | --- | --- | --- | --- |
| 3.5 | 1 | 0.52 | 0.77 | 0.05 | 0.09 |
|  | 2 | 0.31 | 0.85 | 0.03 | 0.13 |
|  | 3 | 0.48 | 0.65 | 0.05 | 0.01 |
|  | 5 | 0.18 | 0.63 | 0.02 | 0.02 |
|  | 6 | 0.28 | 0.72 | 0.02 | 0.04 |
|  | 4 | 0.51 | 0.62 | 0.05 | 0.01 |
|  | 7 | 0.69 | 0.56 | 0.04 | 0.03 |
| 4.0 | 1 | 0.93 | 0.91 | 0.65 | 0.63 |
|  | 3 | 0.76 | 0.84 | 0.34 | 0.32 |
|  | 5 | 0.5 | 0.77 | 0.23 | 0.24 |
|  | 6 | 0.37 | 0.74 | 0.17 | 0.27 |
|  | 4 | 0.84 | 0.74 | 0.37 | 0.24 |
|  | 7 | 0.75 | 0.57 | 0.14 | 0.06 |

**Base:** gpt-4.0, Prompt 3

| **Model** | **Prompt** | **Common correct in base prompt** | **Common correct in comparing prompt** | **Common incorrect in base prompt** | **Common incorrect in comparing prompt** |
| --- | --- | --- | --- | --- | --- |
| 3.5 | 1 | 0.53 | 0.72 | 0.04 | 0.08 |
|  | 2 | 0.33 | 0.82 | 0.03 | 0.13 |
|  | 3 | 0.48 | 0.59 | 0.05 | 0.01 |
|  | 5 | 0.18 | 0.58 | 0.02 | 0.02 |
|  | 6 | 0.29 | 0.68 | 0.02 | 0.04 |
|  | 4 | 0.51 | 0.56 | 0.06 | 0.02 |
|  | 7 | 0.7 | 0.52 | 0.04 | 0.03 |
| 4.0 | 1 | 0.84 | 0.75 | 0.33 | 0.33 |
|  | 2 | 0.84 | 0.76 | 0.32 | 0.34 |
|  | 5 | 0.5 | 0.7 | 0.19 | 0.2 |
|  | 6 | 0.38 | 0.69 | 0.13 | 0.21 |
|  | 4 | 0.84 | 0.68 | 0.36 | 0.24 |
|  | 7 | 0.76 | 0.52 | 0.12 | 0.05 |

**Base:** gpt-4.0, Prompt 4

| **Model** | **Prompt** | **Common correct in base prompt** | **Common correct in comparing prompt** | **Common incorrect in base prompt** | **Common incorrect in comparing prompt** |
| --- | --- | --- | --- | --- | --- |
| 3.5 | 1 | 0.46 | 0.77 | 0.03 | 0.1 |
|  | 2 | 0.28 | 0.86 | 0.02 | 0.14 |
|  | 3 | 0.43 | 0.65 | 0.04 | 0.02 |
|  | 5 | 0.17 | 0.67 | 0.01 | 0.02 |
|  | 6 | 0.25 | 0.74 | 0.01 | 0.03 |
|  | 4 | 0.47 | 0.63 | 0.03 | 0.01 |
|  | 7 | 0.63 | 0.58 | 0.03 | 0.04 |
| 4.0 | 1 | 0.75 | 0.83 | 0.27 | 0.4 |
|  | 2 | 0.74 | 0.84 | 0.24 | 0.37 |
|  | 3 | 0.68 | 0.84 | 0.24 | 0.36 |
|  | 5 | 0.46 | 0.8 | 0.19 | 0.29 |
|  | 6 | 0.34 | 0.76 | 0.13 | 0.32 |
|  | 7 | 0.69 | 0.59 | 0.12 | 0.08 |

**Base:** gpt-4.0, Prompt 5

| **Model** | **Prompt** | **Common correct in base prompt** | **Common correct in comparing prompt** | **Common incorrect in base prompt** | **Common incorrect in comparing prompt** |
| --- | --- | --- | --- | --- | --- |
| 3.5 | 1 | 0.51 | 0.5 | 0.03 | 0.06 |
|  | 2 | 0.33 | 0.59 | 0.02 | 0.1 |
|  | 3 | 0.45 | 0.39 | 0.03 | 0.01 |
|  | 5 | 0.18 | 0.42 | 0.01 | 0.01 |
|  | 6 | 0.27 | 0.46 | 0.02 | 0.03 |
|  | 4 | 0.52 | 0.41 | 0.02 | 0.01 |
|  | 7 | 0.67 | 0.36 | 0.03 | 0.02 |
| 4.0 | 1 | 0.8 | 0.51 | 0.24 | 0.23 |
|  | 2 | 0.77 | 0.5 | 0.24 | 0.23 |
|  | 3 | 0.7 | 0.5 | 0.2 | 0.19 |
|  | 6 | 0.49 | 0.64 | 0.23 | 0.34 |
|  | 4 | 0.8 | 0.46 | 0.29 | 0.19 |
|  | 7 | 0.74 | 0.36 | 0.08 | 0.03 |

**Base:** gpt-4.0, Prompt 6

| **Model** | **Prompt** | **Common correct in base prompt** | **Common correct in comparing prompt** | **Common incorrect in base prompt** | **Common incorrect in comparing prompt** |
| --- | --- | --- | --- | --- | --- |
| 3.5 | 1 | 0.52 | 0.38 | 0.03 | 0.04 |
|  | 2 | 0.36 | 0.49 | 0.02 | 0.05 |
|  | 3 | 0.48 | 0.32 | 0.03 | 0.01 |
|  | 5 | 0.19 | 0.34 | 0.01 | 0 |
|  | 6 | 0.3 | 0.38 | 0.01 | 0.01 |
|  | 4 | 0.5 | 0.3 | 0.03 | 0.01 |
|  | 7 | 0.67 | 0.27 | 0.02 | 0.01 |
| 4.0 | 1 | 0.76 | 0.37 | 0.28 | 0.18 |
|  | 2 | 0.74 | 0.37 | 0.27 | 0.17 |
|  | 3 | 0.69 | 0.38 | 0.21 | 0.13 |
|  | 5 | 0.64 | 0.49 | 0.34 | 0.23 |
|  | 4 | 0.76 | 0.34 | 0.32 | 0.13 |
|  | 7 | 0.72 | 0.27 | 0.12 | 0.03 |

**Base:** gpt-4.0, Prompt 7

| **Model** | **Prompt** | **Common correct in base prompt** | **Common correct in comparing prompt** | **Common incorrect in base prompt** | **Common incorrect in comparing prompt** |
| --- | --- | --- | --- | --- | --- |
| 3.5 | 1 | 0.4 | 0.81 | 0.02 | 0.09 |
|  | 2 | 0.24 | 0.87 | 0.01 | 0.13 |
|  | 3 | 0.36 | 0.66 | 0.03 | 0.02 |
|  | 5 | 0.15 | 0.68 | 0 | 0.01 |
|  | 6 | 0.2 | 0.7 | 0.01 | 0.03 |
|  | 4 | 0.41 | 0.67 | 0.04 | 0.03 |
|  | 7 | 0.65 | 0.71 | 0.02 | 0.06 |
| 4.0 | 1 | 0.57 | 0.75 | 0.05 | 0.14 |
|  | 2 | 0.57 | 0.77 | 0.06 | 0.15 |
|  | 3 | 0.52 | 0.76 | 0.05 | 0.12 |
|  | 5 | 0.36 | 0.75 | 0.03 | 0.09 |
|  | 6 | 0.27 | 0.74 | 0.03 | 0.12 |
|  | 4 | 0.59 | 0.7 | 0.08 | 0.13 |

**APPENDIX 3: Pairwise concordance across all models and prompts using the BIOC-GS corpus**

**Base:** gpt-3.5, Prompt 1

| **Model** | **Prompt** | **Common correct in base prompt** | **Common correct in comparing prompt** | **Common incorrect in base prompt** | **Common incorrect in comparing prompt** |
| --- | --- | --- | --- | --- | --- |
| 3.5 | 2 | 0.42 | 0.97 | 0.14 | 0.65 |
|  | 3 | 0.71 | 0.3 | 0.28 | 0.04 |
|  | 5 | 0.45 | 0.32 | 0.21 | 0.08 |
|  | 6 | 0.55 | 0.34 | 0.17 | 0.05 |
|  | 4 | 0.71 | 0.27 | 0.3 | 0.04 |
|  | 7 | 0.82 | 0.18 | 0.15 | 0.04 |
| 4.0 | 1 | 0.81 | 0.23 | 0.07 | 0.02 |
|  | 2 | 0.8 | 0.22 | 0.1 | 0.02 |
|  | 3 | 0.79 | 0.23 | 0.1 | 0.02 |
|  | 5 | 0.55 | 0.26 | 0.05 | 0 |
|  | 6 | 0.56 | 0.25 | 0.06 | 0.02 |
|  | 4 | 0.87 | 0.2 | 0.11 | 0.02 |
|  | 7 | 0.42 | 0.22 | 0.06 | 0.01 |

**Base:** gpt-3.5, Prompt 2

| **Model** | **Prompt** | **Common correct in base prompt** | **Common correct in comparing prompt** | **Common incorrect in base prompt** | **Common incorrect in comparing prompt** |
| --- | --- | --- | --- | --- | --- |
| 3.5 | 1 | 0.97 | 0.42 | 0.65 | 0.14 |
|  | 3 | 0.81 | 0.15 | 0.24 | 0.01 |
|  | 5 | 0.56 | 0.17 | 0.29 | 0.02 |
|  | 6 | 0.58 | 0.16 | 0.24 | 0.01 |
|  | 4 | 0.75 | 0.12 | 0.18 | 0 |
|  | 7 | 0.81 | 0.08 | 0.06 | 0 |
| 4.0 | 1 | 0.83 | 0.1 | 0.06 | 0 |
|  | 2 | 0.83 | 0.1 | 0.06 | 0 |
|  | 3 | 0.83 | 0.1 | 0.12 | 0.01 |
|  | 5 | 0.56 | 0.11 | 0 | 0 |
|  | 6 | 0.61 | 0.12 | 0.06 | 0 |
|  | 4 | 0.89 | 0.09 | 0.06 | 0 |
|  | 7 | 0.85 | 0.24 | 0.13 | 0.01 |

**Base:** gpt-3.5, Prompt 3

| **Model** | **Prompt** | **Common correct in base prompt** | **Common correct in comparing prompt** | **Common incorrect in base prompt** | **Common incorrect in comparing prompt** |
| --- | --- | --- | --- | --- | --- |
| 3.5 | 1 | 0.3 | 0.71 | 0.04 | 0.28 |
|  | 2 | 0.15 | 0.81 | 0.01 | 0.24 |
|  | 5 | 0.37 | 0.63 | 0.07 | 0.17 |
|  | 6 | 0.44 | 0.65 | 0.11 | 0.2 |
|  | 4 | 0.72 | 0.63 | 0.32 | 0.26 |
|  | 7 | 0.78 | 0.4 | 0.08 | 0.14 |
| 4.0 | 1 | 0.71 | 0.47 | 0.04 | 0.06 |
|  | 2 | 0.71 | 0.47 | 0.05 | 0.08 |
|  | 3 | 0.7 | 0.47 | 0.04 | 0.07 |
|  | 5 | 0.45 | 0.5 | 0.02 | 0.01 |
|  | 6 | 0.44 | 0.47 | 0.03 | 0.07 |
|  | 4 | 0.81 | 0.45 | 0.05 | 0.05 |
|  | 7 | 0.4 | 0.51 | 0.08 | 0.08 |

**Base:** gpt-3.5, Prompt 4

| **Model** | **Prompt** | **Common correct in base prompt** | **Common correct in comparing prompt** | **Common incorrect in base prompt** | **Common incorrect in comparing prompt** |
| --- | --- | --- | --- | --- | --- |
| 3.5 | 1 | 0.27 | 0.71 | 0.04 | 0.3 |
|  | 2 | 0.12 | 0.75 | 0 | 0.18 |
|  | 3 | 0.63 | 0.72 | 0.26 | 0.32 |
|  | 5 | 0.36 | 0.69 | 0.07 | 0.2 |
|  | 6 | 0.41 | 0.68 | 0.1 | 0.22 |
|  | 7 | 0.8 | 0.47 | 0.06 | 0.13 |
| 4.0 | 1 | 0.78 | 0.58 | 0.05 | 0.08 |
|  | 2 | 0.76 | 0.57 | 0.05 | 0.09 |
|  | 3 | 0.76 | 0.58 | 0.03 | 0.06 |
|  | 5 | 0.49 | 0.61 | 0.02 | 0.01 |
|  | 6 | 0.49 | 0.59 | 0.02 | 0.07 |
|  | 4 | 0.86 | 0.53 | 0.05 | 0.07 |
|  | 7 | 0.41 | 0.58 | 0.05 | 0.07 |

**Base:** gpt-3.5, Prompt 5

| **Model** | **Prompt** | **Common correct in base prompt** | **Common correct in comparing prompt** | **Common incorrect in base prompt** | **Common incorrect in comparing prompt** |
| --- | --- | --- | --- | --- | --- |
| 3.5 | 1 | 0.32 | 0.45 | 0.08 | 0.21 |
|  | 2 | 0.17 | 0.56 | 0.02 | 0.29 |
|  | 3 | 0.63 | 0.37 | 0.17 | 0.07 |
|  | 6 | 0.55 | 0.48 | 0.26 | 0.19 |
|  | 4 | 0.69 | 0.36 | 0.2 | 0.07 |
|  | 7 | 0.78 | 0.24 | 0.11 | 0.08 |
| 4.0 | 1 | 0.72 | 0.28 | 0.06 | 0.04 |
|  | 2 | 0.74 | 0.29 | 0.09 | 0.05 |
|  | 3 | 0.71 | 0.29 | 0.09 | 0.06 |
|  | 5 | 0.45 | 0.3 | 0.05 | 0.01 |
|  | 6 | 0.43 | 0.27 | 0.05 | 0.04 |
|  | 4 | 0.85 | 0.28 | 0.09 | 0.04 |
|  | 7 | 0.33 | 0.25 | 0.06 | 0.03 |

**Base:** gpt-3.5, Prompt 6

| **Model** | **Prompt** | **Common correct in base prompt** | **Common correct in comparing prompt** | **Common incorrect in base prompt** | **Common incorrect in comparing prompt** |
| --- | --- | --- | --- | --- | --- |
| 3.5 | 1 | 0.34 | 0.55 | 0.05 | 0.17 |
|  | 2 | 0.16 | 0.58 | 0.01 | 0.24 |
|  | 3 | 0.65 | 0.44 | 0.2 | 0.11 |
|  | 5 | 0.48 | 0.55 | 0.19 | 0.26 |
|  | 4 | 0.68 | 0.41 | 0.22 | 0.1 |
|  | 7 | 0.74 | 0.26 | 0.05 | 0.05 |
| 4.0 | 1 | 0.75 | 0.33 | 0.05 | 0.04 |
|  | 2 | 0.76 | 0.34 | 0.05 | 0.04 |
|  | 3 | 0.75 | 0.35 | 0.03 | 0.03 |
|  | 5 | 0.47 | 0.35 | 0.03 | 0.01 |
|  | 6 | 0.44 | 0.32 | 0.04 | 0.05 |
|  | 4 | 0.85 | 0.32 | 0.06 | 0.04 |
|  | 7 | 0.35 | 0.3 | 0.03 | 0.02 |

**Base:** gpt-3.5, Prompt 7

| **Model** | **Prompt** | **Common correct in base prompt** | **Common correct in comparing prompt** | **Common incorrect in base prompt** | **Common incorrect in comparing prompt** |
| --- | --- | --- | --- | --- | --- |
| 3.5 | 1 | 0.18 | 0.82 | 0.04 | 0.15 |
|  | 2 | 0.08 | 0.81 | 0 | 0.06 |
|  | 3 | 0.4 | 0.78 | 0.14 | 0.08 |
|  | 5 | 0.24 | 0.78 | 0.08 | 0.11 |
|  | 6 | 0.26 | 0.74 | 0.05 | 0.05 |
|  | 4 | 0.47 | 0.8 | 0.13 | 0.06 |
| 4.0 | 1 | 0.61 | 0.77 | 0.08 | 0.07 |
|  | 2 | 0.59 | 0.76 | 0.1 | 0.08 |
|  | 3 | 0.6 | 0.79 | 0.08 | 0.08 |
|  | 5 | 0.35 | 0.76 | 0.04 | 0.01 |
|  | 6 | 0.37 | 0.76 | 0.04 | 0.05 |
|  | 4 | 0.68 | 0.73 | 0.08 | 0.05 |
|  | 7 | 0.3 | 0.74 | 0.1 | 0.06 |

**Base:** gpt-4.0, Prompt 1

| **Model** | **Prompt** | **Common correct in base prompt** | **Common correct in comparing prompt** | **Common incorrect in base prompt** | **Common incorrect in comparing prompt** |
| --- | --- | --- | --- | --- | --- |
| 3.5 | 1 | 0.23 | 0.81 | 0.02 | 0.07 |
|  | 2 | 0.1 | 0.83 | 0 | 0.06 |
|  | 3 | 0.47 | 0.71 | 0.06 | 0.04 |
|  | 5 | 0.28 | 0.72 | 0.04 | 0.06 |
|  | 6 | 0.33 | 0.75 | 0.04 | 0.05 |
|  | 4 | 0.58 | 0.78 | 0.08 | 0.05 |
|  | 7 | 0.77 | 0.61 | 0.07 | 0.08 |
| 4.0 | 2 | 0.94 | 0.95 | 0.67 | 0.65 |
|  | 3 | 0.84 | 0.87 | 0.32 | 0.35 |
|  | 5 | 0.5 | 0.84 | 0.19 | 0.06 |
|  | 6 | 0.48 | 0.78 | 0.11 | 0.17 |
|  | 4 | 0.93 | 0.78 | 0.33 | 0.25 |
|  | 7 | 0.4 | 0.77 | 0.07 | 0.05 |

**Base:** gpt-4.0, Prompt 2

| **Model** | **Prompt** | **Common correct in base prompt** | **Common correct in comparing prompt** | **Common incorrect in base prompt** | **Common incorrect in comparing prompt** |
| --- | --- | --- | --- | --- | --- |
| 3.5 | 1 | 0.22 | 0.8 | 0.02 | 0.1 |
|  | 2 | 0.1 | 0.83 | 0 | 0.06 |
|  | 3 | 0.47 | 0.71 | 0.08 | 0.05 |
|  | 5 | 0.29 | 0.74 | 0.05 | 0.09 |
|  | 6 | 0.34 | 0.76 | 0.04 | 0.05 |
|  | 4 | 0.57 | 0.76 | 0.09 | 0.05 |
|  | 7 | 0.76 | 0.59 | 0.08 | 0.1 |
| 4.0 | 1 | 0.95 | 0.94 | 0.65 | 0.67 |
|  | 3 | 0.83 | 0.86 | 0.32 | 0.36 |
|  | 5 | 0.49 | 0.82 | 0.16 | 0.06 |
|  | 6 | 0.48 | 0.78 | 0.12 | 0.19 |
|  | 4 | 0.93 | 0.77 | 0.33 | 0.26 |
|  | 7 | 0.39 | 0.76 | 0.09 | 0.07 |

**Base:** gpt-4.0, Prompt 3

| **Model** | **Prompt** | **Common correct in base prompt** | **Common correct in comparing prompt** | **Common incorrect in base prompt** | **Common incorrect in comparing prompt** |
| --- | --- | --- | --- | --- | --- |
| 3.5 | 1 | 0.23 | 0.79 | 0.02 | 0.1 |
|  | 2 | 0.1 | 0.83 | 0.01 | 0.12 |
|  | 3 | 0.47 | 0.7 | 0.07 | 0.04 |
|  | 5 | 0.29 | 0.71 | 0.06 | 0.09 |
|  | 6 | 0.35 | 0.75 | 0.03 | 0.03 |
|  | 4 | 0.58 | 0.76 | 0.06 | 0.03 |
|  | 7 | 0.79 | 0.6 | 0.08 | 0.08 |
| 4.0 | 1 | 0.87 | 0.84 | 0.35 | 0.32 |
|  | 2 | 0.86 | 0.83 | 0.36 | 0.32 |
|  | 5 | 0.51 | 0.83 | 0.16 | 0.05 |
|  | 6 | 0.49 | 0.77 | 0.11 | 0.16 |
|  | 4 | 0.95 | 0.77 | 0.37 | 0.26 |
|  | 7 | 0.41 | 0.77 | 0.1 | 0.07 |

**Base:** gpt-4.0, Prompt 4

| **Model** | **Prompt** | **Common correct in base prompt** | **Common correct in comparing prompt** | **Common incorrect in base prompt** | **Common incorrect in comparing prompt** |
| --- | --- | --- | --- | --- | --- |
| 3.5 | 1 | 0.2 | 0.87 | 0.02 | 0.11 |
|  | 2 | 0.09 | 0.89 | 0 | 0.06 |
|  | 3 | 0.45 | 0.81 | 0.05 | 0.05 |
|  | 5 | 0.28 | 0.85 | 0.04 | 0.09 |
|  | 6 | 0.32 | 0.85 | 0.04 | 0.06 |
|  | 4 | 0.53 | 0.86 | 0.07 | 0.05 |
|  | 7 | 0.73 | 0.68 | 0.05 | 0.08 |
| 4.0 | 1 | 0.78 | 0.93 | 0.25 | 0.33 |
|  | 2 | 0.77 | 0.93 | 0.26 | 0.33 |
|  | 3 | 0.77 | 0.95 | 0.26 | 0.37 |
|  | 5 | 0.45 | 0.91 | 0.11 | 0.05 |
|  | 6 | 0.46 | 0.89 | 0.1 | 0.2 |
|  | 7 | 0.38 | 0.87 | 0.08 | 0.08 |

**Base:** gpt-4.0, Prompt 5

| **Model** | **Prompt** | **Common correct in base prompt** | **Common correct in comparing prompt** | **Common incorrect in base prompt** | **Common incorrect in comparing prompt** |
| --- | --- | --- | --- | --- | --- |
| 3.5 | 1 | 0.26 | 0.55 | 0 | 0.05 |
|  | 2 | 0.11 | 0.56 | 0 | 0 |
|  | 3 | 0.5 | 0.45 | 0.01 | 0.02 |
|  | 5 | 0.3 | 0.45 | 0.01 | 0.05 |
|  | 6 | 0.35 | 0.47 | 0.01 | 0.03 |
|  | 4 | 0.61 | 0.49 | 0.01 | 0.02 |
|  | 7 | 0.76 | 0.35 | 0.01 | 0.04 |
| 4.0 | 1 | 0.84 | 0.5 | 0.06 | 0.19 |
|  | 2 | 0.82 | 0.49 | 0.06 | 0.16 |
|  | 3 | 0.83 | 0.51 | 0.05 | 0.16 |
|  | 6 | 0.63 | 0.6 | 0.04 | 0.16 |
|  | 4 | 0.91 | 0.45 | 0.05 | 0.11 |
|  | 7 | 0.41 | 0.47 | 0.01 | 0.03 |

**Base:** gpt-4.0, Prompt 6

| **Model** | **Prompt** | **Common correct in base prompt** | **Common correct in comparing prompt** | **Common incorrect in base prompt** | **Common incorrect in comparing prompt** |
| --- | --- | --- | --- | --- | --- |
| 3.5 | 1 | 0.25 | 0.56 | 0.02 | 0.06 |
|  | 2 | 0.12 | 0.61 | 0 | 0.06 |
|  | 3 | 0.47 | 0.44 | 0.07 | 0.03 |
|  | 5 | 0.27 | 0.43 | 0.04 | 0.05 |
|  | 6 | 0.32 | 0.44 | 0.05 | 0.04 |
|  | 4 | 0.59 | 0.49 | 0.07 | 0.02 |
|  | 7 | 0.76 | 0.37 | 0.05 | 0.04 |
| 4.0 | 1 | 0.78 | 0.48 | 0.17 | 0.11 |
|  | 2 | 0.78 | 0.48 | 0.19 | 0.12 |
|  | 3 | 0.77 | 0.49 | 0.16 | 0.11 |
|  | 5 | 0.6 | 0.63 | 0.16 | 0.04 |
|  | 4 | 0.89 | 0.46 | 0.2 | 0.1 |
|  | 7 | 0.31 | 0.37 | 0.05 | 0.02 |

**Base:** gpt-4.0, Prompt 7

| **Model** | **Prompt** | **Common correct in base prompt** | **Common correct in comparing prompt** | **Common incorrect in base prompt** | **Common incorrect in comparing prompt** |
| --- | --- | --- | --- | --- | --- |
| 3.5 | 1 | 0.22 | 0.42 | 0.01 | 0.06 |
|  | 2 | 0.12 | 0.5 | 0 | 0 |
|  | 3 | 0.51 | 0.4 | 0.08 | 0.08 |
|  | 5 | 0.25 | 0.33 | 0.03 | 0.06 |
|  | 6 | 0.3 | 0.35 | 0.02 | 0.03 |
|  | 4 | 0.58 | 0.41 | 0.07 | 0.05 |
|  | 7 | 0.74 | 0.3 | 0.06 | 0.1 |
| 4.0 | 1 | 0.77 | 0.4 | 0.05 | 0.07 |
|  | 2 | 0.76 | 0.39 | 0.07 | 0.09 |
|  | 3 | 0.77 | 0.41 | 0.07 | 0.1 |
|  | 5 | 0.47 | 0.41 | 0.03 | 0.01 |
|  | 6 | 0.37 | 0.31 | 0.02 | 0.05 |
|  | 4 | 0.87 | 0.38 | 0.08 | 0.08 |

**APPENDIX 4: Top 5 incorrectly extracted HPO IDs across all experiments**

| **Prompt** | **gpt-3.5** | **gpt-4.0** |
| --- | --- | --- |
| 1 | Decreased body weight (HP:0004325)  Intellectual disability, profound (HP:0002187)  Intellectual disability, severe (HP:0010864)  Short stature (HP:0004322)  Nephropathy (HP:0000112) | Poor wound healing (HP:0001058)  Cerebral hamartoma (HP:0009731)  Abnormality of thumb epiphysis (HP:0009599)  Palmar pits (HP:0010610)  Unilateral vestibular schwannoma (HP:0009590) |
| 2 | Decreased body weight (HP:0004325)  Intellectual disability, severe (HP:0010864)  Diabetes insipidus (HP:0000873)  Facial hyperostosis (HP:0005465)  Thin vermilion border (HP:0000233) | Poor wound healing (HP:0001058)  Palmar pits (HP:0010610)  Cerebral hamartoma (HP:0009731)  Unilateral vestibular schwannoma (HP:0009590)  Intellectual disability, severe (HP:0010864) |
| 3 | Intellectual disability, profound (HP:0002187)  Joint hypermobility (HP:0001382)  Microcephaly (HP:0000252)  Hearing impairment (HP:0000365)  Abnormality of the nose (HP:0000366) | Cerebral hamartoma (HP:0009731)  Poor wound healing (HP:0001058)  Neoplasm of the skin (HP:0008069)  Intellectual disability, severe (HP:0010864)  Skin erosion (HP:0200041) |
| 4 | Intellectual disability, profound (HP:0002187)  Joint hypermobility (HP:0001382)  Abnormality of the nervous system (HP:0000707)  Phenotypic abnormality (HP:0000118)  Absent earlobe (HP:0000387) | Poor wound healing (HP:0001058)  Cerebral hamartoma (HP:0009731)  Autosomal dominant inheritance with maternal imprinting (HP:0012275)  Neoplasm of the skin (HP:0008069)  Failure to thrive (HP:0001508 |
| 5 | Nephropathy (HP:0000112)  Intellectual disability, profound (HP:0002187)  Teratoma (HP:0009792)  Abnormality of the nervous system (HP:0000707)  Seizure (HP:0001250) | Poor wound healing (HP:0001058)  Cerebral hamartoma (HP:0009731)  Autosomal dominant inheritance with maternal imprinting (HP:0012275)  Abnormal cellular physiology (HP:0011017)  Occasional neurofibromas (HP:0009595) |
| 6 | Renal cyst (HP:0000107)  Intellectual disability, profound (HP:0002187)  Nephropathy (HP:0000112)  Abnormality of the nervous system (HP:0000707)  Absent earlobe (HP:0000387) | Poor wound healing (HP:0001058)  Autosomal dominant inheritance with maternal imprinting (HP:0012275)  Hypotension (HP:0002615)  Cerebral hamartoma (HP:0009731)  Cataplexy (HP:0002524) |
| 7 | Intellectual disability, profound (HP:0002187)  Abnormality of the nervous system (HP:0000707)  HALLUCINATION (HP:0000000)  Spinal neurofibroma (HP:0009735)  Abnormality of the lower limb (HP:0002814) | Neoplasm of the skin (HP:0008069)  Poor wound healing (HP:0001058)  Unilateral vestibular schwannoma (HP:0009590)  Intellectual disability, severe (HP:0010864)  Cerebral hamartoma (HP:0009731) |
